# Endoscopic gluteal tendon repair reduces complication rates while achieving outcomes comparable to open repair: A multilevel meta‐analysis

**DOI:** 10.1002/ksa.70309

**Published:** 2026-01-31

**Authors:** Nikolai Ramadanov, Maximilian Voss, Ariana Lott, Robert Hable, Robert Prill, Roland Becker, Marko Ostojic, Plamen Penchev, Ingo J. Banke

**Affiliations:** ^1^ Center of Orthopaedics and Traumatology, Brandenburg Medical School University Hospital Brandenburg an der Havel Brandenburg an der Havel Germany; ^2^ Faculty of Health Science Brandenburg, Brandenburg Medical School Theodor Fontane Brandenburg an der Havel Germany; ^3^ Department of Orthopedic Surgery NYU Langone Orthopedic Hospital New York New York USA; ^4^ Faculty of Applied Computer Science, Deggendorf Institute of Technology Deggendorf Germany; ^5^ Sports Traumatology Division, Traumatology Department Draskoviceva University Hospital ‘Sestre Milosrdnice’ Zagreb Croatia; ^6^ Osteon Orthopedics and Sports Medicine Clinic Mostar Bosnia and Herzegovina; ^7^ Faculty of Medicine Medical University of Plovdiv Plovdiv Bulgaria; ^8^ Clinic of Orthopaedics and Sports Orthopaedics, School of Medicine and Health TUM University Hospital, Technical University of Munich Munich Germany; ^9^ AGA‐Society for Arthroscopy and Joint‐Surgery, Hip Committee, c/o Walder Wyss Ltd. Zurich Switzerland

**Keywords:** arthroscopy, endoscopy, gluteal tendon, hip abductor, meta‐analysis, systematic review

## Abstract

**Purpose:**

The purpose of this systematic review and multilevel meta‐analysis was to compare short‐term functional outcomes, pain relief, and complication rates between open and endoscopic gluteal tendon repair, expressed as functional improvement normalized to minimal clinically important difference (MCID) units. The hypothesis was that endoscopic repair would provide comparable clinical efficacy with a lower complication rate compared with open repair.

**Methods:**

PubMed, Embase, CENTRAL and Epistemonikos were searched to 15 October 2025. Eligible primary studies reporting postoperative or change outcomes were synthesized with a frequentist multilevel random‐effects model (inverse variance, restricted maximum likelihood estimation, Hartung–Knapp adjustment).

**Results:**

Thirty‐four studies (1278 patients; 1283 hips) met criteria. Postoperative functional MCID (27 studies; *n* = 1005): overall 9.01 (95% confidence interval [CI]: 8.11–9.91; *I* ^2^ = 100%; *τ* ^2^ = 5.2); open 9.09 (8.12–10.06; six studies; *n* = 398) versus endoscopic 8.96 (8.02–9.90; 17 studies; *n* = 607); no subgroup difference (*p* = 0.63). Change in functional MCID (21 studies; *n* = 718): overall 3.33 (2.86–3.81; *I* ^2^ = 83%; *τ* ^2^ = 0.9); open 3.10 (2.39–3.81; nine studies; *n* = 290) versus endoscopic 3.52 (2.89–4.15; 12 studies; *n* = 428); no difference (*p* = 0.36). Postoperative pain MCID (19 studies; *n* = 867): overall 1.71 (1.27–2.16; *I* ^2^ = 98%; *τ* ^2^ = 0.9); open 1.58 (1.03–2.13; nine studies; *n* = 349) versus endoscopic 1.81 (1.31–2.32; 11 studies; *n* = 518); no difference (*p* = 0.38). Change in pain MCID (19 studies; *n* = 798): overall –1.99 (–2.74 to –1.24; *I* ^2^ = 99%; *τ* ^2^ = 2.5); open –1.82 (–2.96 to –0.69; nine studies; *n* = 349) versus endoscopic –2.12 (–3.18 to –1.09; 11 studies; *n* = 449); no difference (*p* = 0.68). Overall complications (25 studies; *n* = 804): 58/804 (0.07; 95% CI: 0.05–0.11; *I* ^2^ = 53%; *τ* ^2^ = 0.8); open 33/371 (0.08; 0.04–0.16) versus endoscopic 25/433 (0.07; 0.04–0.12); no difference (*p* = 0.70).

**Conclusion:**

Open and endoscopic gluteal tendon repair provide clinically meaningful short‐term improvements in function and pain, with no relevant differences in efficacy when interpreted using MCID units. These findings support both techniques in clinical practice and favour endoscopic repair when available due to its less invasive nature.

**Level of Evidence:**

Level III, systematic review and meta‐analysis of predominantly Level III studies.

**Abbreviations**
AMSTAR: A MeaSurement Tool to Assess systematic Reviews
CI: confidence interval
HHS: Harris Hip Score
HOOS: Hip disability and Osteoarthritis Outcome Score
HOS‐ADL: Hip Outcome Score—Activities of Daily Living
HOS‐SSS: Hip Outcome Score—Sports Specific Subscale
iHOT‐12: International Hip Outcome Tool—12 Items
MCID: minimal clinically important difference
mHHS: modified Harris Hip Score
OHS: Oxford Hip Score
PRISMA: Preferred Reporting Items for Systematic Reviews and Meta‐Analyses
PROM: patient‐reported outcome measure
PROSPERO: International Prospective Register of Systematic Reviews
RCT: randomized controlled trial
RoB: Risk of Bias
ROBINS: Risk Of Bias In Non‐randomised Studies of Interventions
SD: standard deviation
TITAN: Transparency in the reporting of artificial intelligence
VAS: Visual Analogue Scale
WOMAC: Western Ontario and McMaster Universities Osteoarthritis Index

## INTRODUCTION

Gluteus medius and minimus tendon tears—often termed the ‘rotator cuff tears of the hip’—are increasingly recognized as a cause of chronic lateral hip pain and abductor dysfunction, particularly in middle‐aged to older women [25, 33]. They affect up to ~10% of native hips over 60 and up to 28% of osteoarthritic hips undergoing total hip arthroplasty (THA), with even higher prevalence after (revision) THA, where they contribute to persistent postoperative pain [7, 11, 26, 49]. Gluteal pathology is associated with inferior THA outcomes, even when asymptomatic [60].

Surgical repair is indicated when conservative treatment fails, and imaging confirms tendon detachment [30]. Traditionally, repair has been performed via an open approach using anchors or transosseous tunnels, analogous to rotator cuff repair [2, 22, 70]. More recently, endoscopic repair has emerged [20], with several described technique modifications including biological augmentation such as microfracture [29].

Current evidence suggests comparable functional outcomes between techniques, including pain relief, satisfaction, and strength recovery [13, 43], while open repair is associated with higher complication rates (7%–8% vs. ~0.7% endoscopic) [13, 33]. Open repair may remain preferable for retracted full‐thickness tears requiring mobilization or augmentation, with similarly low re‐tear rates (3%–4%) [33]. However, most available data derive from retrospective case series (Level IV), with few direct comparisons and no randomized trials [2, 13, 20]. Prior reviews reported functional improvement with both approaches but fewer complications after arthroscopy [33], while emphasizing the need for higher‐quality evidence [13]. Looney et al. [34] demonstrated that high‐grade fatty infiltration limits functional improvement but not pain relief, underscoring the influence of muscle quality and the limited scope of existing analyses.

Despite multiple previous reviews, the literature remains limited by small sample sizes, descriptive synthesis, heterogeneous patient‐reported outcome measure (PROM) instruments and a lack of clinically interpretable pooled effect estimates. Importantly, no prior synthesis has applied a multilevel meta‐analytic framework or standardized outcomes using minimal clinically important difference (MCID) units to harmonize heterogeneous PROMs.

The purpose of this study was to compare short‐term functional outcomes, pain relief, and complication rates between open and endoscopic gluteal tendon repair using a multilevel meta‐analysis. It was hypothesized that endoscopic repair achieves comparable clinical efficacy in function and pain, while being associated with lower complication rates compared with open repair.

## METHODS

### Definition

Several terms are used in the literature to describe injuries of the lateral hip tendons, including gluteus medius tendon, gluteus minimus tendon, hip abductor tendon, gluteal tendon and hip rotator cuff. As these terms refer to the same anatomical structures involved in abductor function, the term ‘gluteal tendon’ was used consistently throughout this study.

### Reporting standards and protocol registration

The peer‐reviewed study protocol [57] was registered in the International Prospective Register of Systematic Reviews (PROSPERO) on 11 July 2025 (CRD420251088765). This meta‐analysis adhered to the Preferred Reporting Items for Systematic Reviews and Meta‐Analyses (PRISMA) 2020 guidelines [47], with the completed checklist provided as Table S1. The study further followed the 2025 Transparency in the Reporting of Artificial Intelligence (TITAN) guideline [1], with the completed checklist provided as Table S2. An author self‐assessment using A MeaSurement Tool to Assess systematic Reviews (AMSTAR) 2 [63] was performed by two independent reviewers to document adherence to critical methodological domains; the completed checklist is provided in Table S3.

### Search strategy and study selection

A comprehensive database search was conducted in PubMed, Embase, CENTRAL (Cochrane Library) and Epistemonikos, covering studies published up to 15 October 2025. Boolean logic was applied using database‐specific syntax, including: ((gluteus medius) OR (gluteus minimus) OR (gluteal tendon) OR (hip abductor)) AND ((arthroscopy) OR (endoscopy) OR (endoscopic) OR (arthroscopic) OR (open)). No restrictions were applied regarding language or publication year. Two reviewers (M. V. and A. L.) independently performed a two‐stage screening process (title/abstract, then full text). Disagreements were resolved by a third reviewer (N. R.). Inter‐rater reliability was assessed using Cohen's kappa (*κ*).

### Eligibility criteria

Eligible studies included randomized controlled trials (RCTs), prospective or retrospective observational studies and case series. Case reports, narrative reviews and editorials were excluded. Exclusion criteria were: (1) insufficient description of surgical technique and (2) absence of relevant functional or pain‐related outcomes. Importantly, eligibility was determined by the availability of outcomes assessed at short‐term follow‐up (≤24 months), irrespective of the maximum follow‐up duration reported. If both short‐ and long‐term outcomes were available, only short‐term data were extracted; studies reporting exclusively long‐term outcomes were excluded.

### Data extraction

Data were independently extracted by two reviewers (M. V. and A. L.), with discrepancies resolved by consensus with a third reviewer (N. R.). Extracted variables included author, year, country, sample size, demographics, study design, risk of bias, follow‐up and outcome measures. Missing standard deviations were addressed by contacting authors, estimating from reported ranges (range/4) or imputing using established methods [74]. PROM scoring systems were reviewed to ensure directional consistency. Visual analogue scale (VAS) scores were standardized to a 0–10 scale, and Western Ontario and McMaster Universities Osteoarthritis Index scores were inverted so that higher values consistently indicated better outcomes.

### MCID normalization

PROM values were standardized by expressing outcomes in MCID units, calculated by dividing observed means and standard deviations by the respective MCID of each instrument. This approach enabled harmonization across heterogeneous PROMs and pooling into a single functional MCID outcome [55].

### Primary outcome analysis

Primary outcomes comprised postoperative functional outcomes and change scores, expressed in MCID units; postoperative pain outcomes and their change scores, also expressed in MCID units and overall complication rates. Postoperative functional MCID reflects functional PROM values normalized to the MCID, whereas change in functional MCID represents the pre‐ to postoperative change expressed relative to the MCID (not a change of the MCID itself). Analogous definitions applied to postoperative pain MCID and change in pain MCID.

### Secondary outcome analyses

Secondary analyses included PROM‐specific evaluations (International Hip Outcome Tool—12 Items [iHOT‐12], Hip Outcome Score—Sports Specific Subscale [HOS‐SSS], Hip Outcome Score—Activities of Daily Living [HOS‐ADL], Hip disability and Osteoarthritis Outcome Score [HOOS], modified Harris Hip Score [mHHS] and Oxford Hip Score [OHS]), performed to assess consistency across instruments and support interpretation of the MCID‐based pooled outcomes rather than to provide independent comparisons.

### Risk of bias assessment

Risk of bias was independently assessed by two reviewers (M. V. and A. L.) using Risk of Bias (RoB) 2 for randomized trials [64] and ROBINS‐I for non‐randomized studies [65], with adjudication by a third reviewer (N. R.). Publication bias was assessed using Begg's test [5] and funnel plot inspection [66].

### Statistical analysis

Treatment effects were analysed using a frequentist multilevel random‐effects meta‐analysis with inverse variance weighting, restricted maximum likelihood estimation and Hartung–Knapp adjustment [56]. Continuous outcomes are reported as means with 95% confidence intervals (CIs). Subgroup analyses compared open versus endoscopic techniques, and heterogeneity was quantified using Higgins' *I* ^2^ statistic. All analyses were performed using the R packages meta and metafor.

## RESULTS

### Systematic literature search

A total of 503 records were screened by title and abstract after removal of 1020 duplicates, with high inter‐reviewer agreement (*κ* = 0.98). Forty‐seven full‐text articles [3, 4, 6, 8, 9, 10, 12, 14, 15, 16, 17, 18, 19, 21, 23, 24, 27, 28, 31, 32, 35, 36, 37, 38, 39, 40, 41, 42, 44, 45, 46, 48, 50, 51, 52, 53, 54, 58, 59, 61, 62, 67, 68, 69, 71, 72, 73] were assessed for eligibility with full agreement (*κ* = 1.0). Thirteen studies were excluded because treatment groups could not be differentiated [8, 9, 23, 39, 40, 61, 71], relevant outcomes were not reported [14, 21] or only long‐term outcomes were available [35, 48, 50, 54].

**Figure 1:**
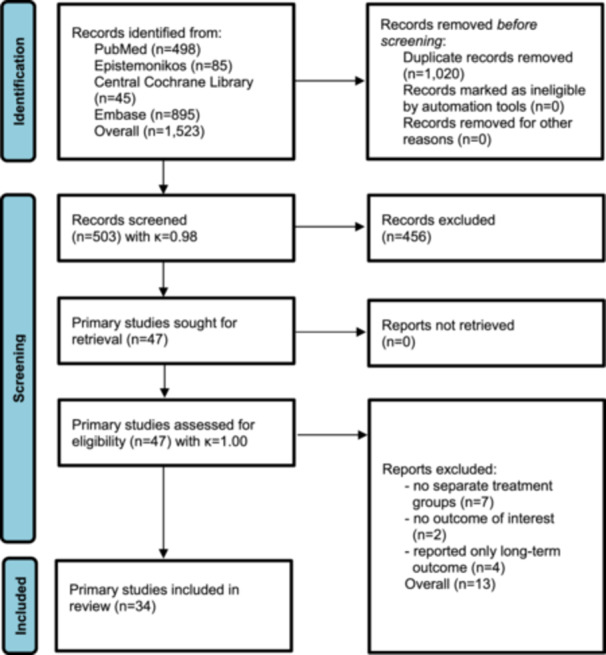
PRISMA flow diagram. PRISMA, Preferred Reporting Items for Systematic Reviews and Meta‐Analyses.

Ultimately, 34 primary studies [3, 4, 6, 10, 12, 15, 16, 17, 18, 19, 24, 27, 28, 31, 32, 36, 37, 38, 41, 42, 44, 45, 46, 51, 52, 53, 58, 59, 62, 67, 68, 69, 72, 73] including 1278 patients (1283 hips) were included in the multilevel meta‐analysis (Figure 1).

### Patient characteristics

The open repair group included 14 studies [3, 10, 15, 17, 24, 31, 36, 37, 38, 52, 53, 59, 62, 73] with 490 patients (491 hips), 6.3% male, mean age 54.2 years and mean body mass index (BMI) 28.7 kg/m^2^.

The endoscopic group comprised 23 studies [4, 6, 12, 16, 18, 19, 24, 27, 28, 32, 37, 38, 41, 42, 44, 45, 46, 51, 58, 67, 68, 69, 72] with 788 patients (792 hips), 9.6% male, mean age 57.7 years and mean BMI 27.3 kg/m^2^.

Further study and patient details are summarized in Table 1. Preoperative PROMs did not differ significantly between groups (Figures [Link], [Link], [Link], [Link], [Link], [Link], [Link], [Link], Table S4).

**Table 1:** Study and patient characteristics.

Author	Origin	Journal	Study design	LoE	Operation	Patients, *N*	Hips, *N*	Age, years ± SD (range)	Male sex, *N* (%)	BMI, kg/m^2^ ± SD (range)	Follow‐up duration (months)^a^
Barrera M et al. 2021 [3]	Switzerland	*Journal of Hip Preservation Surgery*	Retrospective case series	IV	Mini‐open	14	14	62.4 ± 18.0	1 (7.0)		14.3 ± 7.0 (6‐24)
Bauwens PH et al. 2021 [4]	France	Orthopaedics & Traumatology: Surgery & Research	Retrospective case series	IV	Endoscopic	6	6	73.3	2 (33.3)	28.2	>24
Bitar AC et al. 2023 [6]	Brazil	*The Archives of Bone and Joint Surgery*	Retrospective case series	IV	Endoscopic	16	16	65.0 (32.0–73.0)	1 (6.0)		42 (12–131)
Bucher TA et al. 2013 [10]	United Kingdom	*HIP International*	Prospective case series	IV	Open	22	22	62.0 (49.0–74.0)	3 (13.6)		12
Chandrasekaran S et al. 2015 [12]	USA	*The Journal of Bone and Joint Surgery*	Retrospective case series	IV	Endoscopic	34	34	57.0 (20.1–78.8)	2 (6.0)	27.9 (19.2–40.4)	27.2 (24.4–45.9)
					Endoscopic	10	10	54.4 (20.1–71.1)	0 (0.0)	28.5 (21.4–34.5)	
					Endoscopic	24	24	58.7 (41.3–78.8)	2 (8.0)	28.0 (19.2–40.4)	
Day MA et al. 2022 [15]	USA	*Journal of Hip Preservation Surgery*	Prospective case series	IV	Open	9	9	60.3 ± 9.3	1 (11.1)	29.4 ± 6.3	6
Della Rocca F et al. 2023 [16]	Italy	*Knee Surgery, Sports Traumatology, Arthroscopy*	Retrospective case series	IV	Endoscopic	22	22	58.6 (52.0–69.0)	4 (18.2)	28.5 (20.6–34.3)	42 ± 14.5 (24–72)
Derksen A et al. 2022 [17]	Germany	*Journal of Experimental Orthopaedics*	Retrospective case series	IV	Open	43	43	65.2 ± 11.3 (41.3–85.1)	11 (25.6)		22.8 ± 9.7 (9–46)
Domb BG et al. 2013 (1) [18]	USA	*The American Journal of Sports Medicine*	Prospective case series	IV	Endoscopic	15	15	57.8 (44.0–74.0)	1 (6.7)	26.2 (19.9–32.6)	27.9 (24–37)
					Endoscopic	6	6	57.3 (49.0–70.0)		25.5 (24.0–26.9)	
					Endoscopic	9	9	58.1 (44.0–74.0)		26.5 (19.9–32.6)	
Domb BG et al. 2024 (2) [19]	USA	*Arthroscopy: The Journal of Arthroscopic and Related Surgery*	Prospective case series	IV	Endoscopic	11	11	60.1 ± 10.1 (46.2–74.8)	1 (9.1)	25.4 ± 4.9 (24.0–34.2)	124.4 ± 55.1 (120.0–153.1)
Gilat R et al. 2024 [24]	Israel	*Knee Surgery, Sports Traumatology, Arthroscopy*	Retrospective case series	IV	Open	38	38	NR	NR	NR	37.2 ± 16.8
					Endoscopic	69	69	NR	NR	NR	
Horner NS et al. 2023 [27]	USA	*The American Journal of Sports Medicine*	Retrospective cohort study	III	Endoscopic	31	31	50.8 ± 7.3	4 (12.9)	27.9 ± 5.2	26.2 ± 5.2
Kirby D et al. 2020 [28]	USA	*Arthroscopy: The Journal of Arthroscopic and Related Surgery*	Retrospective case series	IV	Endoscopic	20	20	51.3 ± 11.9	5 (21.0)	25.3 ± 3.9	28.8 ± 11.3
					Endoscopic	12	12	54.8 ± 11.3	4 (27.0)	25.1 ± 4.0	
					Endoscopic	8	8	46.0 ± 11.4	1 (12.0)	25.5 ± 4.0	
Lange J et al. 2024 [31]	Denmark	*Danish Medical Journal*	Retrospective observational study	IV	Open	67	68	59.0 (56.0–61.0)	0 (0.0)	28.0 (27.0–29.0)	12
Larson JH et al. 2024 [32]	USA	*The Orthopaedic Journal of Sports Medicine*	Retrospective propensity‐matched cohort study	III	Endoscopic	32	32	50.6 ± 8.0	3 (9.4)	27.8 ± 6.2	24
					Endoscopic	32	32	52.7 ± 8.9	1 (3.1)	28.9 ± 6.8	
Maldonado DR et al. 2020 [36]	USA	*The Orthopaedic Journal of Sports Medicine*	Retrospective case series	IV	Open	36	36	65.2 ± 12.7 (57.8–73.7)	5 (13.9)	29.0 ± 5.0 (26.4–31.2)	40.8 ± 26.19 (24.16–46.20)
Maslaris A et al. 2020 (1) [37]	Greece	*Knee Surgery, Sports Traumatology, Arthroscopy*	Retrospective cohort study	III	Open	23	23	68.0 ± 8.2	3 (13.0)	29.0 ± 5.2	20.3 ± 12.5 (3–63)
					Open	12	12	67.0 ± 8.2	4 (33.0)	29.7 ± 7.3	
					Endoscopic	10	10	59.0 ± 10.0	3 (30.0)	25.8 ± 1.8	
Maslaris A et al. 2022 (2) [38]	USA	*Archives of Orthopaedic and Trauma Surgery*	Retrospective cohort study	III	Endoscopic	10	38	64.0 ± 12.2	1 (10.0)	28.2 ± 6.5	20.9 ± 12.5
			retrospectiv		Open	6	23	63.0 ± 8.6	3 (50.0)	27.7 ± 4.2	
			retrospectiv		Open	12	16	69.5 ± 11.3	4 (25.0)	30.2 ± 7.6	
Meghpara MB et al. 2020 (1) [41]	USA	*Arthroscopy: The Journal of Arthroscopic and Related Surgery*	Retrospective case series	IV	Endoscopic	37	37	51.5 ± 10.5 (17.2–74.8)	3 (8.1)	27.7 ± 13.1 (19.4–40.4)	73.4 (60.0–105.1)
Meghpara MB et al. 2021 (2) [42]	USA	*Arthroscopy, Sports Medicine, and Rehabilitation*	Retrospective comparative cohort study	III	Endoscopic	23	23	62.7 ± 10.1 (36.9–74.4)	1 (4.3)	29.9 ± 4.2 (24.0–84.5)	38.3 ± 19.2 (24.0–84.5)
Nadeau NJ et al. 2024 [44]	USA	*Arthroscopy: The Journal of Arthroscopic and Related Surgery*	Retrospective comparative cohort study	III	Endoscopic	26	26	57.0 ± 12.0	6 (13.0)	29.0 ± 5.0	≥24
					Endoscopic	38	38	59.0 ± 12.0	2 (5.0)	28.0 ± 5.0	
Nazal MR et al. 2020 [45]	USA	*Arthroscopy: The Journal of Arthroscopic and Related Surgery*	Prospective case series	IV	Endoscopic	13	15	66.9 ± 9.0	3 (20.0)	28.8 ± 3.9	31.2 ± 10.9 (24–60)
Okoroha KR et al. 2019 [46]	USA	*The American Journal of Sports Medicine*	Retrospective case series	IV	Endoscopic	60	60	57.9 ± 9.9	5 (8.3)	27.6 ± 6.1	24 (22–24)
Prabhavalkar ON et al. 2023 [51]	USA	*The American Journal of Sports Medicine*	Propensity‐matched comparative cohort study	III	Endoscopic	48	48	53.3 ± 9.8 (30.9–72.2)	4 (8.0)	26.7 ± 4.6 (18.8–40.7)	38.5 ± 15.7 (24.0–72.8)
					Endoscopic	48	48	54.3 ± 9.0 (32.1–76.0)	3 (6.0)	27.1 ± 4.3 (17.5–38.8)	58.8 ± 17.2 (24.0–88.8)
Quickenborne D et al. 2025 [52]	Belgium	*International Journal of Surgery Case Reports*	Prospective case series	IV	Open	42	42		4 (9.5)		12 (6–12)
Quinn M et al. 2024 [53]	USA	*Arthroscopy, Sports Medicine, and Rehabilitation*	Retrospective case series	IV	Mini‐open	61	61	61.4 ± 1.3	2 (3.2)		25.9 ± 1.13 (24–30.9)
Rice MW et al. 2022 (1) [58]	USA	*The American Journal of Sports Medicine*	Retrospective case series	IV	Endoscopic	46	46	59.1 ± 8.9	6 (13.0)	27.3 ± 6.9	72.0
Rice MW et al. 2023 (2) [59]	USA	*Journal of Hip Preservation Surgery*	Retrospective case series	IV	Open	25	25	69.0 ± 6.8	1 (4.0)	26.9 ± 5.0	37.2 (30.0–64.8)
Schröder JH et al. 2018 [62]	Germany	*Der Orthopäde*	Retrospective cohort study	IV	Open	12	12	58.0 (43.0–75.0)	0 (0.0)		19 (12–46)
Thaunat M et al. 2011 (1) [67]	France	*Orthopaedics & Traumatology: Surgery & Research*	Retrospective case series	IV	Endoscopic	4	4	68.5 (64.0–79.0)			6
Thaunat M et al. 2018 (2) [68]	France	*Arthroscopy: The Journal of Arthroscopic and Related Surgery*	Retrospective case series	IV	Endoscopic	20	22	66.0 ± 8.0 (45.0–82.0)	3 (15.0)	27.6 ± 4.9	31.7 ± 7.6 (24–47)
Thaunat M et al. 2021 (3) [69]	France	*Arthroscopy: The Journal of Arthroscopic and Related Surgery*	Retrospective case series	IV	Endoscopic	46	46	62.7 ± 9.0 (43.0–82.0)	3 (6.5)	25.7 ± 4.0 (19.0–34.0)	46.7 ± 15 (24–72
Voos JE et al. 2009 [72]	USA	*The American Journal of Sports Medicine*	Prospective case series	IV	Endoscopic	10	10	50.4 (33.0–66.0)	2 (20.0)		25 (19–38)
Walsh MJ et al. 2011 [73]	Australia	*Journal of Arthroplasty*	Retrospective cohort study	IV	Open	72	72	62 (36.0–88.0)	5 (7.0)		12
Total						1278	1283	59.6 (46.0–73.3)	131 (10.2)	27.6 (25.1–30.2)	32.22 (6.0–124.4)
Open surgery group						490	491	54.2 (59.0–69.5	31 (6.3)	28.7 (26.9–30.2)	38.4 (6.0–40.8)
Endoscopic surgery group						788	792	57.7 (46.0–73.3)	76 (9.6)	27.3 (25.1–29.9)	29.0 (6.0–124.4)

Abbreviations: BMI, body mass index; LoE, level of evidence; SD, standard deviation.

^a^ Although some studies report longer follow‐up periods, only short‐term outcomes (≤24 months postoperatively) were included in the quantitative synthesis.

### Quality assessment

Among the 34 included studies, five showed moderate risk of bias [27, 32, 42, 44, 51], whereas 29 were rated as serious risk of bias [3, 4, 6, 10, 12, 15, 16, 17, 18, 19, 24, 28, 31, 36, 37, 38, 41, 45, 46, 52, 53, 58, 59, 62, 67, 68, 69, 72, 73] (Table 2). Funnel plots indicated variable publication bias. Minimal asymmetry was observed for postoperative functional MCID (Figure S9) and pain MCID (Figure S11), little bias for change in functional MCID (Figure S10), moderate asymmetry for change in pain MCID (Figure S12) and pronounced asymmetry for complications, suggesting relevant publication bias (Figure S13).

**Table 2:** Risk of bias assessment using the ROBINS‐I tool for non‐RCTs.

Author	Bias due to confounding	Bias in selection of participants	Bias in classification of interventions	Bias due to deviations from intended interventions	Bias due to missing data	Bias in measurement of outcomes	Bias in selection of the reported result	Overall risk of bias
Barrera M et al. 2021 [3]	Serious	Moderate	Low	Low	Low	Moderate	Moderate	Serious
Bauwens PH et al. 2021 [4]	Serious	Moderate	Low	Low	Low	Moderate	Moderate	Serious
Bitar AC et al. 2023 [6]	Serious	Moderate	Low	Low	Moderate	Moderate	Moderate	Serious
Bucher TA et al. 2013 [10]	Serious	Moderate	Low	Low	Low	Moderate	Moderate	Serious
Chandrasekaran S et al. 2015 [12]	Serious	Moderate	Low	Low	Moderate	Moderate	Moderate	Serious
Day MA et al. 2022 [15]	Serious	Moderate	Low	Low	Moderate	Moderate	Moderate	Serious
Della Rocca F et al. 2023 [16]	Serious	Moderate	Low	Low	Moderate	Moderate	Moderate	Serious
Derksen A et al. 2022 [17]	Serious	Moderate	Low	Low	Moderate	Moderate	Moderate	Serious
Domb BG et al. 2013 (1)[18]	Serious	Moderate	Low	Low	Low	Moderate	Moderate	Serious
Domb BG et al. 2024 (2) [19]	Serious	Moderate	Low	Low	Low	Moderate	Moderate	Serious
Gilat R et al. 2024 [24]	Serious	Moderate	Low	Low	Moderate	Moderate	Moderate	Serious
Horner NS et al. 2023 [27]	Moderate	Moderate	Low	Low	Low	Moderate	Moderate	Moderate
Kirby D et al. 2020 [28]	Serious	Moderate	Low	Low	Moderate	Moderate	Moderate	Serious
Lange J et al. 2024 [31]	Serious	Moderate	Low	Low	Low	Moderate	Moderate	Serious
Larson JH et al. 2024 [32]	Moderate	Moderate	Low	Low	Low	Moderate	Moderate	Moderate
Maldonado DR et al. 2020 [36]	Serious	Moderate	Low	Low	Moderate	Moderate	Moderate	Serious
Maslaris A et al. 2020 (1) [37]	Serious	Moderate	Low	Low	Moderate	Moderate	Moderate	Serious
Maslaris A et al. 2022 (2) [38]	Serious	Moderate	Low	Low	Moderate	Moderate	Moderate	Serious
Meghpara MB et al. 2020 (1) [41]	Serious	Moderate	Low	Low	Moderate	Moderate	Moderate	Serious
Meghpara MB et al. (2) 2021 [42]	Moderate	Moderate	Low	Low	Low	Moderate	Moderate	Moderate
Nadeau NJ et al. 2024 [44]	Moderate	Moderate	Low	Low	Low	Moderate	Moderate	Moderate
Nazal MR et al. 2020 [45]	Serious	Moderate	Low	Low	Low	Moderate	Moderate	Serious
Okoroha KR et al. 2019 [46]	Serious	Moderate	Low	Low	Moderate	Moderate	Moderate	Serious
Prabhavalkar ON et al. 2023 [51]	Moderate	Moderate	Low	Low	Low	Moderate	Moderate	Moderate
Quickenborne D et al. 2025 [52]	Serious	Moderate	Low	Low	Moderate	Moderate	Moderate	Serious
Quinn M et al. 2024 [53]	Serious	Moderate	Low	Low	Moderate	Moderate	Moderate	Serious
Rice MW et al. 2022 (1) [58]	Serious	Moderate	Low	Low	Low	Moderate	Moderate	Serious
Rice MW et al. 2023 (2) [59]	Serious	Moderate	Low	Low	Low	Moderate	Moderate	Serious
Schröder JH et al. 2018 [62]	Serious	Moderate	Low	Low	Moderate	Moderate	Moderate	Serious
Thaunat M et al. 2011 (1) [67]	Serious	Moderate	Low	Low	Low	Moderate	Moderate	Serious
Thaunat M et al. 2018 (2) [68]	Serious	Moderate	Low	Low	Low	Moderate	Moderate	Serious
Thaunat M et al. 2021 (3) [69]	Serious	Moderate	Low	Low	Low	Moderate	Moderate	Serious
Voos JE et al. 2009 [72]	Serious	Moderate	Low	Low	Low	Moderate	Moderate	Serious
Walsh MJ et al. 2011 [73]	Serious	Moderate	Low	Low	Moderate	Moderate	Moderate	Serious

Abbreviations: RCTs, randomized controlled trials; ROBINS‐I, Risk Of Bias In Non‐randomized Studies of Interventions.

### Multilevel meta‐analysis of primary outcomes

#### Postoperative functional MCID

**Figure 2:**
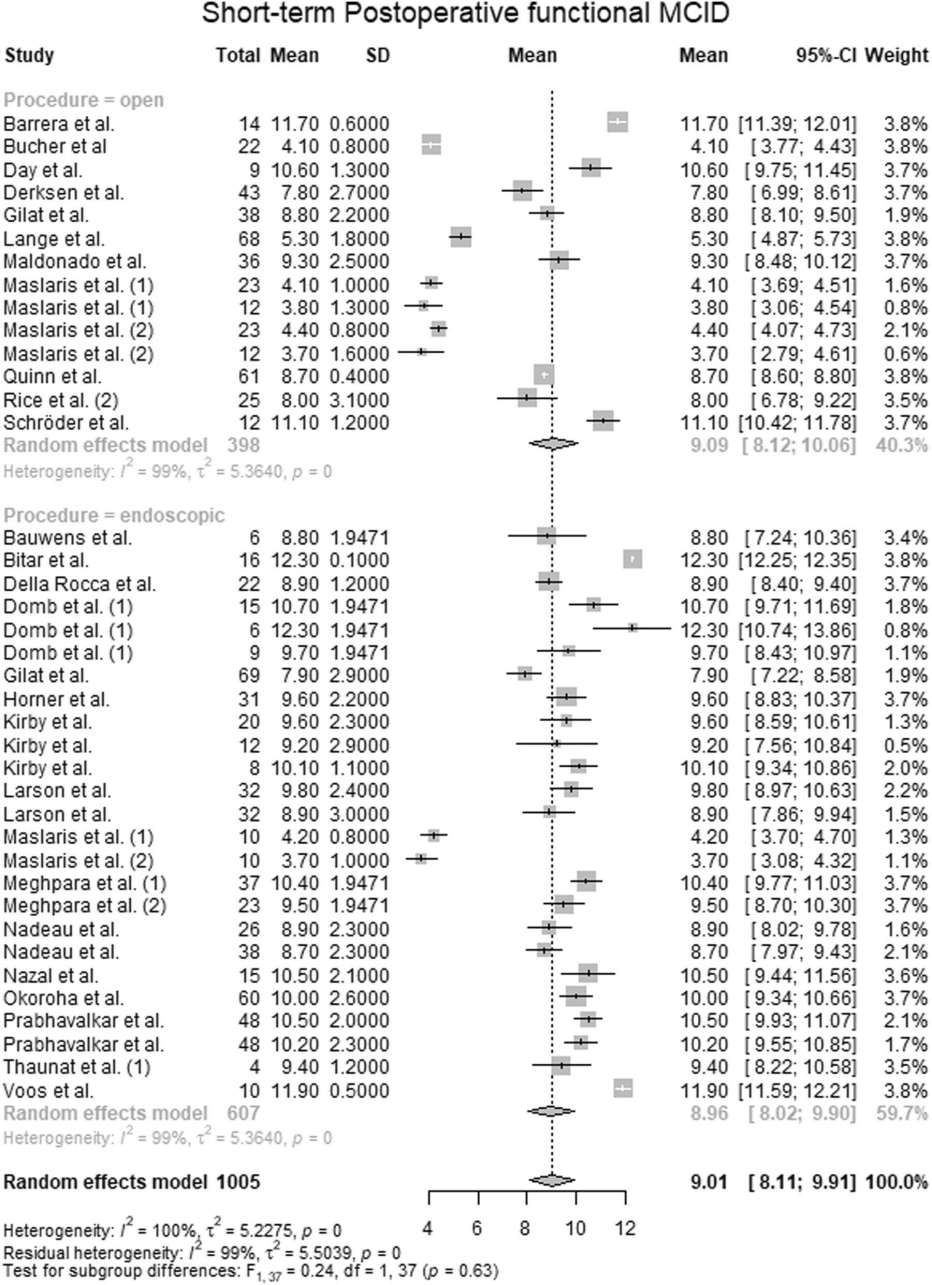
Forest plot of the postoperative functional MCID. CI, confidence interval; MCID, minimal clinically important difference; SD, standard deviation.

Data from 1005 patients from 27 primary studies [3, 4, 6, 10, 15, 16, 17, 18, 24, 27, 28, 31, 32, 36, 37, 38, 41, 42, 44, 45, 46, 51, 53, 59, 62, 67, 72] were pooled (Figure 2, Table S4), with the open surgery group consisting of 398 patients from 12 primary studies [3, 10, 15, 17, 24, 31, 36, 37, 38, 53, 59, 62] and the endoscopic surgery group consisting of 607 patients from 18 primary studies [4, 6, 16, 18, 24, 27, 28, 32, 37, 38, 39, 42, 44, 45, 46, 51, 67, 72]. The mean postoperative functional MCID of the entire patient group was 9.01 points (mean: 9.01; CIs: 8.11–9.91; *I* ^2^ = 100%; *τ* ^2^ = 5.2; *p* = 0.00). The mean postoperative functional MCID of the open surgery group was 9.09 points (mean: 9.09; CIs: 8.12–10.06; *I* ^2^ = 99%; *τ* ^2^ = 5.4; *p* = 0.00). The mean postoperative functional MCID of the endoscopic surgery group was 8.96 points (mean: 8.96; CIs: 8.02–9.90; *I* ^2^ = 99%; *τ* ^2^ = 5.4; *p* = 0.00). The test for subgroup differences showed no statistically significant differences in postoperative functional MCID between the open surgery group and the endoscopic surgery group (*F* = 0.24; df = 1.0; *p* = 0.63).

#### Change in functional MCID

**Figure 3:**
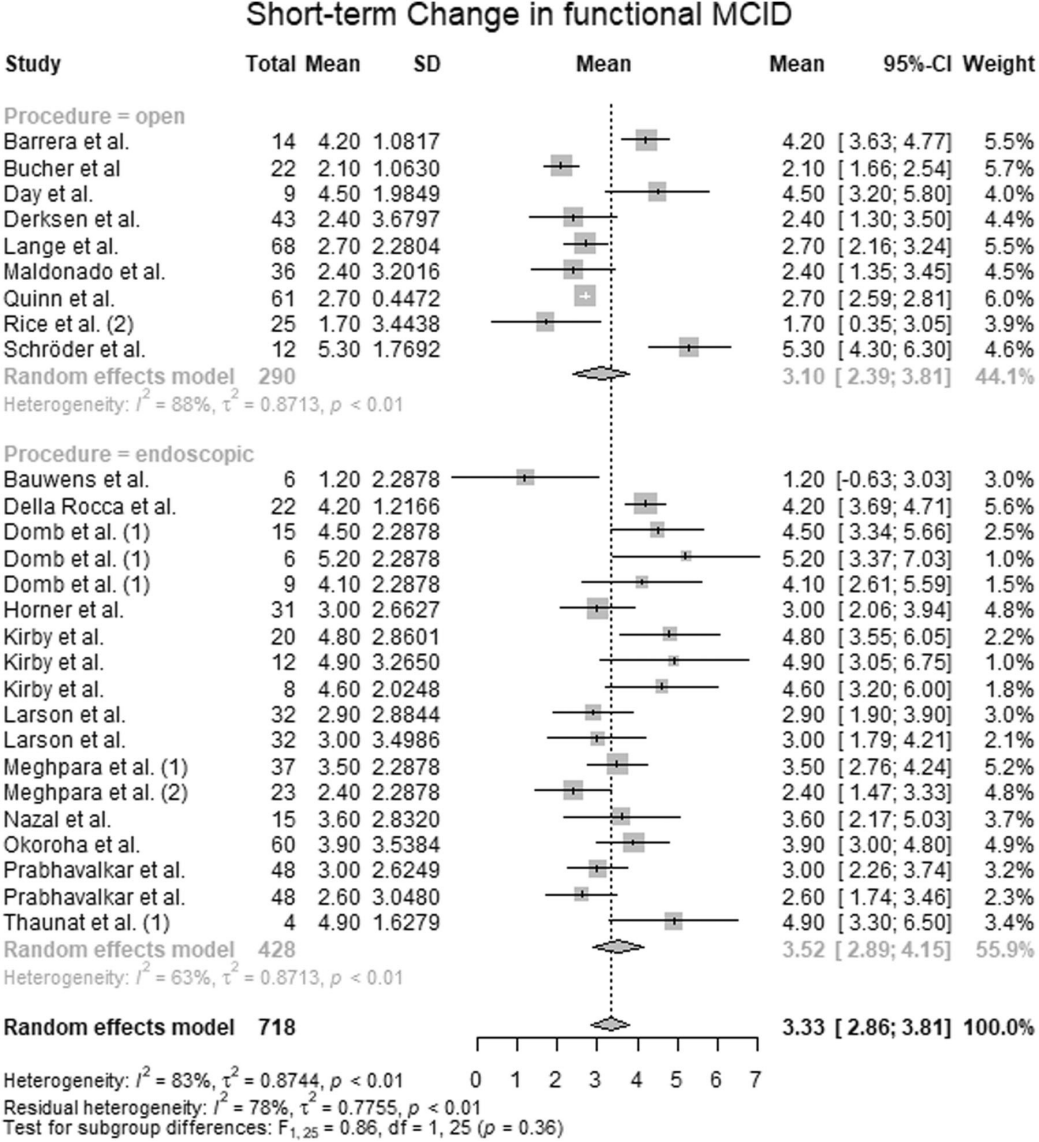
Forest plot of the change in functional MCID. CI, confidence interval; MCID, minimal clinically important difference; SD, standard deviation.

Data from 718 patients from 21 primary studies [3, 4, 10, 15, 16, 17, 18, 27, 28, 31, 32, 36, 41, 42, 45, 46, 51, 53, 59, 62, 67] were pooled (Figure 3, Table S4), with the open surgery group consisting of 290 patients from 9 primary studies [3, 10, 15, 17, 31, 36, 53, 59, 62] and the endoscopic surgery group consisting of 428 patients from 12 primary studies [4, 16, 18, 27, 28, 32, 41, 42, 45, 46, 51, 67]. The mean change in functional MCID of the entire patient group was 3.33 points (mean: 3.33; CIs: 2.86–3.81; *I* ^2^ = 83%; *τ* ^2^ = 0.9; *p* < 0.01). The mean change in functional MCID of the open surgery group was 3.10 points (mean: 3.10; CIs: 2.39–3.81; *I* ^2^ = 88%; *τ* ^2^ = 0.9; *p* < 0.01). The mean change in functional MCID of the endoscopic surgery group was 3.52 points (mean: 3.52; CIs: 2.89–4.15; *I* ^2^ = 63%; *τ* ^2^ = 0.9; *p* < 0.01). The test for subgroup differences showed no statistically significant differences in change in functional MCID between the open surgery group and the endoscopic surgery group (*F* = 0.86; df = 1.0; *p* = 0.36).

#### Postoperative pain MCID

**Figure 4:**
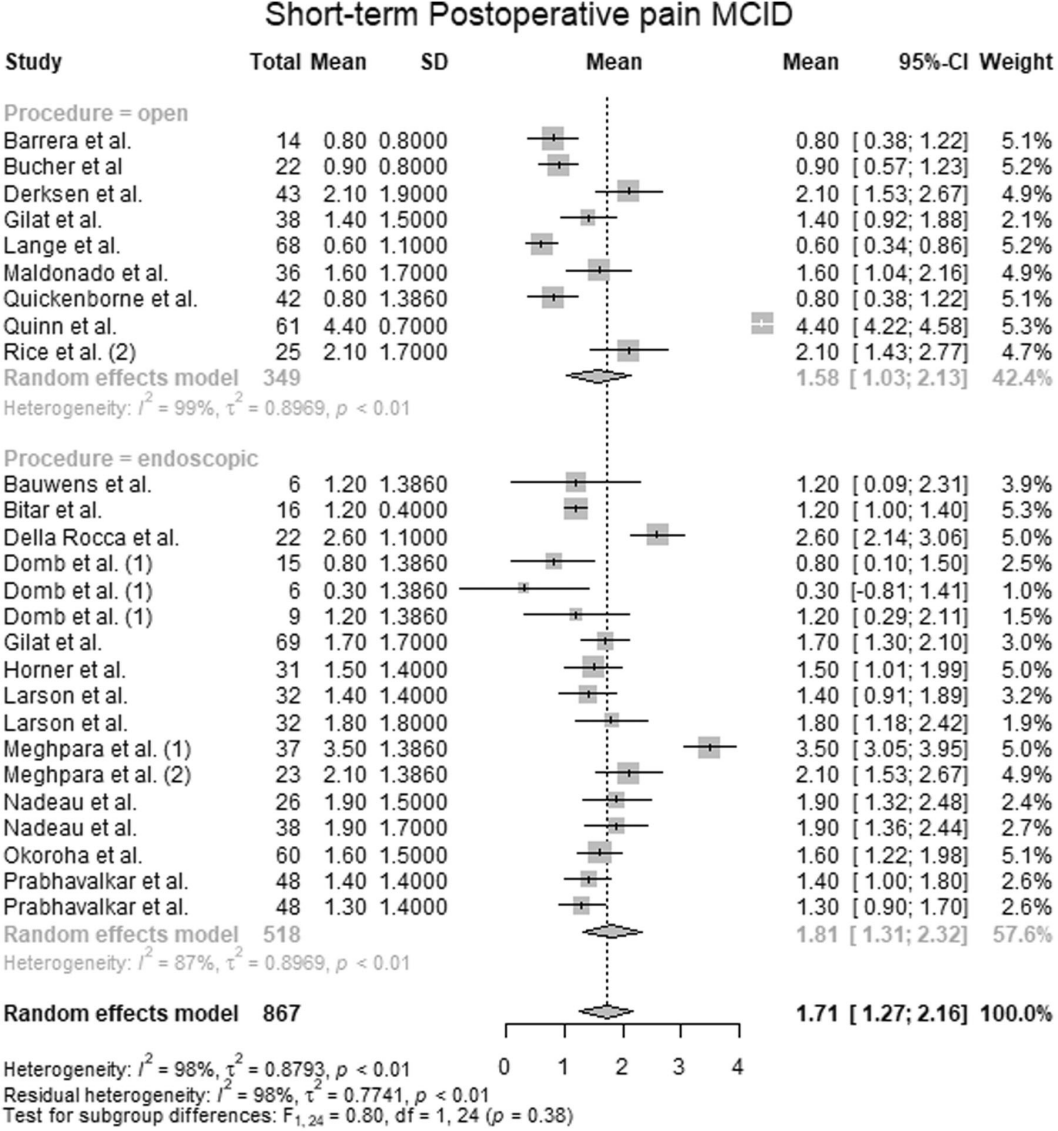
Forest plot of the postoperative pain MCID. CI, confidence interval; MCID, minimal clinically important difference; SD, standard deviation.

Data from 867 patients from 20 primary studies [3, 4, 6, 10, 16, 17, 18, 24, 27, 31, 32, 36, 41, 42, 44, 46, 51, 52, 53, 59] were pooled (Figure 4, Table S4), with the open surgery group consisting of 349 patients from 9 primary studies [3, 10, 17, 24, 31, 36, 52, 53, 59] and the endoscopic surgery group consisting of 518 patients from 12 primary studies [4, 6, 16, 18, 24, 27, 32, 41, 42, 44, 46, 51]. The mean postoperative pain MCID of the entire patient group was 1.71 points (mean: 1.71; CIs: 1.27–2.16; *I* ^2^ = 98%; *τ* ^2^ = 0.9; *p* < 0.01). The mean postoperative pain MCID of the open surgery group was 1.58 points (mean: 1.58; CIs: 1.03–2.13; *I* ^2^ = 99%; *τ* ^2^ = 0.9; *p* < 0.01). The mean postoperative pain MCID of the endoscopic surgery group was 1.81 points (mean: 1.81; CIs: 1.31–2.32; *I* ^2^ = 87%; *τ* ^2^ = 0.9; *p* < 0.01). The test for subgroup differences showed no statistically significant differences in postoperative pain MCID between the open surgery group and the endoscopic surgery group (*F* = 0.80; df = 1.0; *p* = 0.38).

#### Change in pain MCID

**Figure 5:**
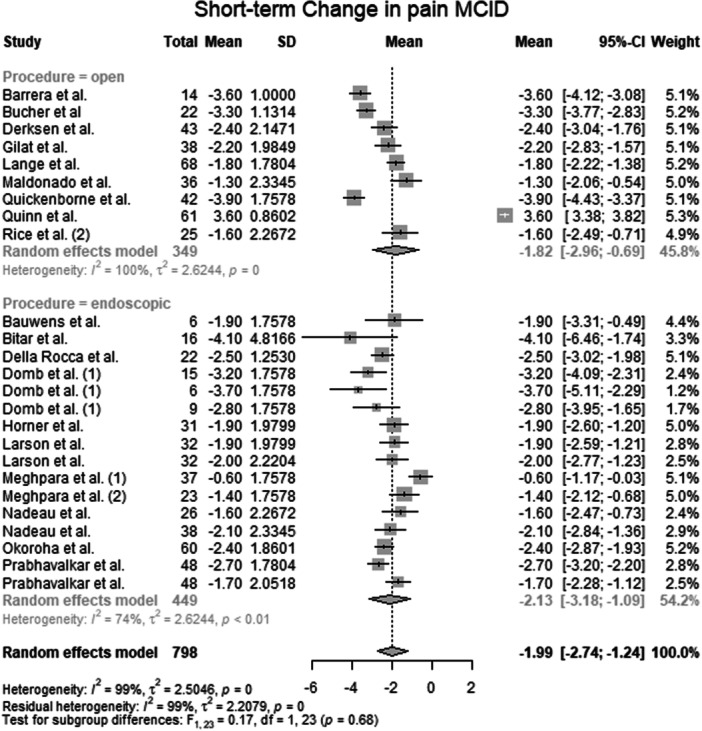
Forest plot of the change in pain MCID. CI, confidence interval; MCID, minimal clinically important difference; SD, standard deviation.

Data from 798 patients from 20 primary studies [3, 4, 6, 10, 16, 17, 18, 24, 27, 31, 32, 36, 41, 42, 44, 46, 51, 52, 53, 59] were pooled (Figure 5, Table S4), with the open surgery group consisting of 349 patients from 9 primary studies [3, 10, 17, 24, 31, 36, 52, 53, 59] and the endoscopic surgery group consisting of 449 patients from 11 primary studies [4, 6, 16, 18, 27, 32, 41, 42, 44, 46, 51]. The mean change in pain MCID of the entire patient group was −1.99 points (mean: −1.99; CIs: −2.74 to −1.24; *I* ^2^ = 99%; *τ* ^2^ = 2.5; *p* = 0.00). The mean change in pain MCID of the open surgery group was −1.82 points (mean: −1.82; CIs: −2.96 to −0.69; *I* ^2^ = 100%; *τ* ^2^ = 2.6; *p* = 0.00). The mean change in pain MCID of the endoscopic surgery group was −2.12 points (mean: −2.12; CIs: −3.18 to −1.09; *I* ^2^ = 74%; *τ* ^2^ = 2.6; *p* < 0.01). The test for subgroup differences showed no statistically significant differences in change in pain MCID between the open surgery group and the endoscopic surgery group (*F* = 0.17; df = 1.0; *p* = 0.68).

#### Overall complications

**Figure 6:**
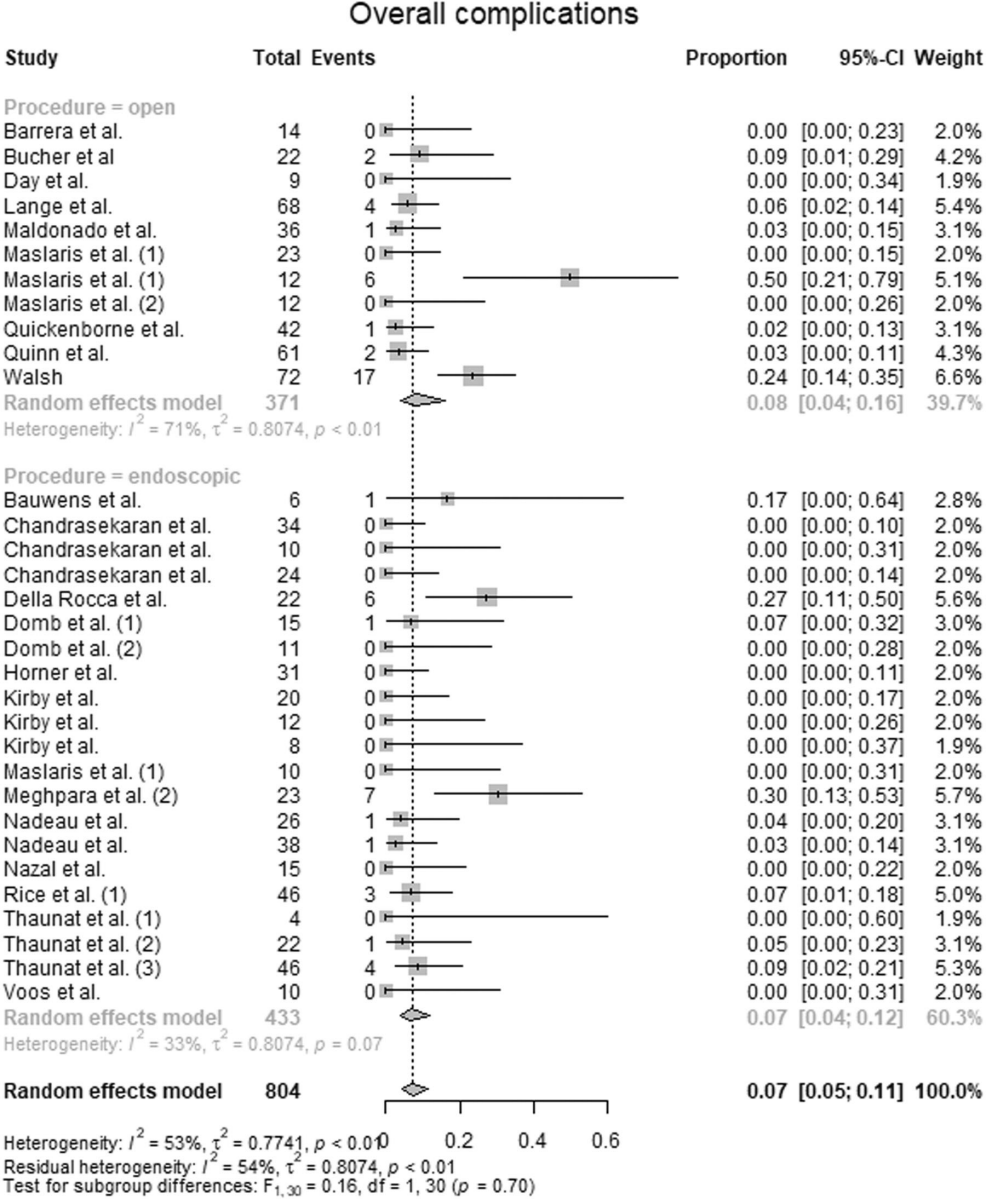
Forest plot of the overall complications. CI, confidence interval.

Data from 804 patients from 25 primary studies [3, 4, 10, 12, 15, 16, 18, 19, 27, 28, 31, 36, 37, 38, 41, 44, 45, 52, 53, 59, 67, 68, 69, 72, 73] were pooled (Figure 6, Table S4), with the open surgery group consisting of 371 patients from 9 primary studies [3, 10, 15, 31, 36, 37, 38, 52, 53, 73] and the endoscopic surgery group consisting of 433 patients from 16 primary studies [4, 12, 16, 18, 19, 27, 28, 38, 41, 44, 45, 58, 67, 68, 69, 72]. The pooled proportion of overall complications for the entire patient group was 0.07 (95% CI: 0.05–0.11, *I* ^2^ = 53%, *τ* ^2^ = 0.8; *p* < 0.01), corresponding to a crude event rate of 58/804. For the open surgery group, the pooled proportion of overall complications was 0.08 (95% CI: 0.04–0.16, *I* ^2^ = 71%, *τ* ^2^ = 0.8; *p* < 0.01), with a crude event rate of 33/371 (0.09). For the endoscopic surgery group, the pooled proportion of overall complications was 0.07 (95% CI: 0.04–0.12, *I* ^2^ = 33%, *τ* ^2^ = 0.8; *p* = 0.07), with a crude event rate of 25/433 (0.06). The test for subgroup differences showed no statistically significant differences in overall complications between the open surgery group and the endoscopic surgery group (*F* = 0.16; df = 1.0; *p* = 0.70).

#### Multilevel meta‐analysis of secondary outcomes

The postoperative PROMs iHOT‐12, HOS‐SSS, HOS‐ADL and HOOS, and the change in mHHS, iHOT‐12, HOS‐SSS, HOS‐ADL and OHS showed no statistically significant differences between the open surgery group and the endoscopic surgery group (Figures [Link], [Link], [Link], [Link], [Link], [Link], [Link], [Link], [Link]). The funnel plots of primary and secondary outcomes are presented in Figures [Link], [Link], [Link], [Link], [Link], [Link], [Link], [Link], [Link], [Link].

## DISCUSSION

The most important finding of the present study was that both open and endoscopic gluteal tendon repair result in clinically meaningful improvements in function and pain, with no relevant differences in efficacy between techniques. However, endoscopic repair was associated with lower complication rates and may therefore be preferred in most clinical scenarios when arthroscopic expertise is available. These findings have direct clinical implications, suggesting that surgical approach selection should be guided primarily by invasiveness and complication risk rather than expectations of superior functional recovery. In contrast to earlier descriptive reviews based on limited patient numbers [13], the present study provides a quantitative synthesis of a substantially larger evidence base using MCID‐based interpretation, offering clinically meaningful guidance for decision‐making.

The central distinction between open and endoscopic repair appears to relate to safety rather than efficacy. Both techniques achieved clinically relevant improvements in patient‐reported outcomes, underscoring that gluteal tendon repair itself effectively addresses pain and dysfunction. The open approach showed a non‐significant trend toward higher adverse event rates, particularly wound‐related complications, infections and postoperative haematomas, which may negatively affect recovery, increase healthcare utilization and reduce patient satisfaction.

The consistency of functional and pain improvements across both groups suggests that the pathophysiological target—tendon reattachment—is adequately addressed by either technique. The primary differentiator is therefore the extent of surgical exposure. Endoscopic repair minimizes soft tissue trauma and preserves vascularity, which may partly explain the lower complication rates, an important consideration in the typically older, predominantly female patient population with higher comorbidity burden.

The findings of this multilevel meta‐analysis align with prior systematic reviews [13, 33], which similarly reported equivalent functional outcomes but a more favourable complication profile for endoscopic repair. Longstaffe et al. [33] and Chandrasekaran et al. [13] reported low and comparable retear rates (approximately 3%–4%), reinforcing that structural healing is not substantially technique‐dependent. More recent cohort studies [36, 43] further support these observations, demonstrating sustained improvements in strength, gait and patient satisfaction.

By pooling a substantially larger dataset and applying a multilevel meta‐analytic framework, the present study extends prior work with greater statistical precision. MCID‐based normalization allowed harmonization of heterogeneous PROM instruments, addressing a major limitation of earlier analyses. Funnel plot asymmetry—particularly for complications—suggests possible publication bias, but the overall pattern consistently indicates higher morbidity without superior efficacy after open repair.

From a clinical standpoint, both techniques remain valid options for gluteal tendon tears, with endoscopic repair favoured when technically feasible. Reduced complication rates may translate into shorter hospital stays, fewer reoperations and lower overall costs. However, endoscopic repair is technically demanding, may require longer operative time and depends on arthroscopic expertise, particularly during the learning curve.

Open repair retains an important role in complex scenarios, including massive or retracted tears, poor tissue quality or when concomitant procedures necessitate open exposure. Surgical decision‐making should therefore remain individualized, incorporating tear characteristics, patient comorbidities and surgeon experience. Shared decision‐making is essential, as patients can be counselled that functional outcomes are comparable, while perioperative risk profiles differ.

This study has several limitations. The evidence base consists predominantly of retrospective case series (Level IV), with few prospective or comparative studies and no RCTs. Heterogeneity in patient selection, concomitant procedures and rehabilitation protocols may have influenced outcomes. Despite MCID‐based standardization, residual bias from pooling heterogeneous PROMs cannot be excluded. Importantly, tear size, tear pattern, and tissue quality were inconsistently reported, introducing potential selection bias, as larger or more complex tears may be preferentially treated with open repair. The absence of non‐operative comparator studies further limits contextualization of surgical outcomes. Finally, the predominance of studies with serious risk of bias [3, 4, 6, 10, 12, 15, 16, 17, 18, 19, 24, 28, 31, 36, 37, 38, 41, 45, 46, 52, 53, 58, 59, 62, 67, 68, 69, 72, 73] and evidence of publication bias—particularly for complications—necessitate cautious interpretation.

In daily clinical practice, these findings support informed surgical counselling. When both approaches are feasible, endoscopic repair may be preferred due to lower complication risk without sacrificing functional recovery or pain relief, while open repair remains appropriate for complex tear patterns or compromised tissue quality.

## CONCLUSION

Open and endoscopic gluteal tendon repair provide clinically meaningful short‐term improvements in function and pain, with no relevant differences in efficacy. Endoscopic repair is associated with lower complication rates and may be considered when arthroscopic expertise is available. This multilevel meta‐analysis adds quantitative evidence from a larger dataset using multilevel modelling and MCID‐based interpretation, offering clearer guidance for clinical decision‐making.

## AUTHOR CONTRIBUTIONS

Nikolai Ramadanov, Maximilian Voss and Ariana Lott performed the literature search, the data extraction and the risk of bias assessment. Robert Hable and Nikolai Ramadanov conducted the statistical calculations. Robert Hable and Nikolai Ramadanov created all figures and tables. Nikolai Ramadanov wrote the manuscript. Ingo J. Banke, Robert Prill, Plamen Penchev, Marko Ostojic and Roland Becker supervised the work.

## CONFLICT OF INTEREST STATEMENT

The authors declare no conflicts of interest.

## ETHICS STATEMENT

Ethics approval was not required as this study is a multilevel meta‐analysis of previously published primary studies.

## Supporting information

Supporting information.

Supporting information.

Supporting information.

Supporting information.

Supporting information.

Supporting information.

Supporting information.

Supporting information.

Supporting information.

Supporting information.

Supporting information.

Supporting information.

Supporting information.

Supporting information.

Supporting information.

Supporting information.

Supporting information.

Supporting information.

Supporting information.

Supporting information.

Supporting information.

Supporting information.

Supporting information.

Supporting information.

Supporting information.

Supporting information.

Supporting information.

Supporting information.

Supporting information.

Supporting information.

Supporting information.

Supporting information.

Supporting information.

Supporting information.

Supporting information.

Supporting information.

Supporting information.

## Data Availability

Available from the corresponding author upon reasonable request.

## References

[1] Agha RA , Mathew G , Rashid R , Kerwan A , Al‐Jabir A , Sohrabi C , et al.; TITAN Group . Transparency in the reporting of artificial intelligence—the TITAN guideline. Premier J Sci. 2025;10:100082.

[2] Alpaugh K , Chilelli BJ , Xu S , Martin SD . Outcomes after primary open or endoscopic abductor tendon repair in the hip: a systematic review of the literature. Arthroscopy. 2015;31(3):530–540. 25442666 10.1016/j.arthro.2014.09.001

[3] Barrera M , Bothorel H , Poultsides L , Christofilopoulos P . Short‐term outcomes following mini‐open repair of chronic gluteus medius tendon tears using a double‐row technique. J Hip Preserv Surg. 2021;8(2):202–208. 35145719 10.1093/jhps/hnab060 PMC8825688

[4] Bauwens PH , Haidar I , Thaunat M . Endoscopic transfer of gluteus maximus and tensor fasciae latae for massive gluteus medius tear: preliminary results. Orthop Traumatol Surg Res. 2021;107(8):102927. 10.1016/j.otsr.2021.102927 33845176

[5] Begg CB , Mazumdar M . Operating characteristics of a rank correlation test for publication bias. Biometrics. 1994;50(4):1088–1101. 7786990

[6] Bitar AC , Guimarães JB , Marques R , de Castro Trindade CA , Filho AGO , Nico MAC , et al. Clinical and radiological results after endoscopic treatment for gluteal tendon injuries with a minimum follow‐up of 12 months. Arch Bone Joint Surg. 2023;11(10):641–648. 37873531 10.22038/ABJS.2023.70495.3304 PMC10590485

[7] Brethouwer DA , Brown ML , McCauley JC , Bugbee WD , Chang EY , Lombardi AF , et al. What is the prevalence of hip abductor pathology in patients undergoing total hip arthroplasty? Arthroplast Today. 2024;31:101601. 10.1016/j.artd.2024.101601 39811777 PMC11732240

[8] Browning RB , Fenn TW , Allahabadi S , Rice MW , Swindell HW , Ebersole JW , et al. Three‐grade magnetic resonance imaging‐based gluteus medius and/or minimus tear classification system provides excellent inter‐rater reliability. Arthrosc Sports Med Rehabil. 2023;5(3):e773–e782. 37388882 10.1016/j.asmr.2023.04.004 PMC10300604

[9] Browning RB , Fenn TW , Allahabadi S , Vogel MJ , Chapman RS , Beals C , et al. Open and endoscopic gluteus medius and/or minimus repair achieves clinical success regardless of tear grade: high‐grade fatty infiltration portends worse outcomes. Arthroscopy. 2025;41(4):966–977.e2. 38844013 10.1016/j.arthro.2024.05.021

[10] Bucher TA , Darcy P , Ebert JR , Smith A , Janes G . Gluteal tendon repair augmented with a synthetic ligament: surgical technique and a case series. HIP Int. 2014;24(2):187–193. 24186680 10.5301/hipint.5000093

[11] Bunker TD , Esler CNA , Leach WJ . Rotator‐cuff tear of the hip. J Bone Joint Surg Br. 1997;79(4):618–620. 9250749 10.1302/0301-620x.79b4.7033

[12] Chandrasekaran S , Gui C , Hutchinson MR , Lodhia P , Suarez‐Ahedo C , Domb BG . Outcomes of endoscopic gluteus medius repair: study of thirty‐four patients with minimum two‐year follow‐up. J Bone Jt Surg. 2015;97(16):1340–1347. 10.2106/JBJS.N.01229 26290085

[13] Chandrasekaran S , Lodhia P , Gui C , Vemula SP , Martin TJ , Domb BG . Outcomes of open versus endoscopic repair of abductor muscle tears of the hip: a systematic review. Arthroscopy. 2015;31(10):2057–2067.e2. 26033462 10.1016/j.arthro.2015.03.042

[14] Chandrasekaran S , Vemula SP , Gui C , Suarez‐Ahedo C , Lodhia P , Domb BG . Clinical features that predict the need for operative intervention in gluteus medius tears. Orthop J Sports Med. 2015;3(2):2325967115571079. 10.1177/2325967115571079 26535383 PMC4555614

[15] Day MA , Hancock KJ , Selley RS , Swartwout EL , Dooley M , Shamrock AG , et al. Repair of gluteus medius tears with bioinductive collagen patch augmentation: initial evaluation of safety and imaging. J Hip Preserv Surg. 2022;9(3):185–190. 35992027 10.1093/jhps/hnac031 PMC9389905

[16] Della Rocca F , Di Francia V , Giuffrida A , Rosolani M , D'Ambrosi R , D'Addona A . Satisfactory results after endoscopic gluteus medius repair combined with selective gluteus maximus reflected tendon release for the treatment of a full‐thickness tear of gluteus medius. Knee Surg Sports Traumatol Arthrosc. 2023;31(5):2038–2045. 36066574 10.1007/s00167-022-07140-x PMC10090025

[17] Derksen A , Lonnemann E , Budde S , Becker O , Wirries N , Haertlé M , et al. Clinical results after open gluteus medius repair in single‐row technique. J Exp Orthop. 2022;9(1):55. 35689698 10.1186/s40634-022-00483-x PMC9188632

[18] Domb BG , Botser I , Giordano BD . Outcomes of endoscopic gluteus medius repair with minimum 2‐year follow‐up. Am J Sports Med. 2013;41(5):988–997. 23524152 10.1177/0363546513481575

[19] Domb BG , Owens JS , Maldonado DR , Harris WT , Perez‐Padilla PA , Sabetian PW . Favorable and durable outcomes at 10‐year follow‐up after endoscopic gluteus medius repair with concomitant hip arthroscopy. Arthroscopy. 2024;40(8):2215–2224. 37967732 10.1016/j.arthro.2023.10.049

[20] Domb BG , Nasser RM , Botser IB . Partial‐thickness tears of the gluteus medius: rationale and technique for trans‐tendinous endoscopic repair. Arthroscopy. 2010;26(12):1697–1705. 20951538 10.1016/j.arthro.2010.06.002

[21] Domb BG , Prabhavalkar ON , Padmanabhan S , Carbone AD . Predictors of clinical outcomes after hip arthroscopy: 10‐year follow‐up analysis of 1038 patients. Am J Sports Med. 2024;52(8):2029–2036. 38869367 10.1177/03635465241254076

[22] Flynn ME , Beason DP , Bartush KC , Ryan MK , Emblom BA . Endoscopic gluteus medius repair replicates open, knotless repair with similar cyclic loading properties: a cadaveric study. Arthrosc Sports Med Rehabil. 2022;4(2):e617–e622. 35494267 10.1016/j.asmr.2021.12.002 PMC9042893

[23] Gilat R , Vogel MJ , Alvero AB , Malloy P , Nho SJ . Use and effectiveness of physical therapy after primary gluteus medius and/or minimus repair: a duration‐ and session‐sensitive analysis. Am J Sports Med. 2025;53(7):1671–1680. 40289421 10.1177/03635465251334768

[24] Gilat R , Vogel MJ , Kazi O , Nho SJ . Defining clinically significant outcome thresholds for the Patient‐Reported Outcomes Measurement Information System (PROMIS) at 2 years after gluteus medius and/or minimus repair. Orthop J Sports Med. 2024;12(11):23259671241281746. 10.1177/23259671241281746 39525349 PMC11544753

[25] Hartigan DE , Perets I , Ho SW , Walsh JP , Yuen LC , Domb BG . Endoscopic repair of partial‐thickness undersurface tears of the abductor tendon: clinical outcomes with minimum 2‐year follow‐up. Arthroscopy. 2018;34(4):1193–1199. 29305287 10.1016/j.arthro.2017.10.022

[26] Hecht CJ , Lavu MS , Kaelber DC , Homma Y , Kamath AF . Association between abductor tears and hip pathology: a nationwide large cohort study. J Orthop. 2024;53:140–146. 38601894 10.1016/j.jor.2024.03.036 PMC11002529

[27] Horner NS , Chapman RS , Larson JH , Nho SJ . Results of endoscopic labral repair with concomitant gluteus medius and/or minimus repair compared with outcomes of labral repair alone: a matched comparative cohort analysis at minimum 2‐year follow‐up. Am J Sports Med. 2023;51(7):1818–1825. 37103484 10.1177/03635465231166708

[28] Kirby D , Fried JW , Bloom DA , Buchalter D , Youm T . Clinical outcomes after endoscopic repair of gluteus medius tendon tear using a knotless technique with a 2‐year minimum follow‐up. Arthroscopy. 2020;36(11):2849–2855. 32721548 10.1016/j.arthro.2020.07.022

[29] Kocaoglu B , Paksoy AE , Cerciello S , Ollivier M , Seil R , Safran M . Arthroscopic repair of the hip abductor musculotendinous unit: the effect of microfracture on clinical outcomes. Am J Sports Med. 2021;49(6):1570–1577. 33793365 10.1177/0363546521999678

[30] Lachiewicz PF . Abductor tendon tears of the hip: evaluation and management. J Am Acad Orthop Surg. 2011;19(7):385–391. 21724917 10.5435/00124635-201107000-00001

[31] Lange J , Lund B , Spoorendonk K , Bohn MB . Open surgical repair of hip abductor tendon tears. Dan Med J. 2024;71(3):A08230526. 38445316 10.61409/A08230526

[32] Larson JH , Brusalis CM , Allahabadi S , Fenn TW , Chapman RS , Browning RB , et al. Outcomes of isolated endoscopic gluteal tendon repair compared with concomitant endoscopic gluteal tendon repair and arthroscopic hip labral repair: a propensity‐matched analysis with minimum 2‐year follow‐up. Orthop J Sports Med. 2024;12(2):23259671231215340. 10.1177/23259671231215340 38379577 PMC10878227

[33] Longstaffe R , Dickerson P , Thigpen CA , Shanley E , Kissenberth MJ , Folk J , et al. Both open and endoscopic gluteal tendon repairs lead to functional improvement with similar failure rates: a systematic review. J ISAKOS. 2021;6(1):28–34. 33833043 10.1136/jisakos-2020-000474

[34] Looney AM , Bodendorfer BM , Donaldson ST , Browning RB , Chahla JA , Nho SJ . Influence of fatty infiltration on hip abductor repair outcomes: a systematic review and meta‐analysis. Am J Sports Med. 2022;50(9):2568–2580. 34495797 10.1177/03635465211027911

[35] Makridis KG , Lequesne M , Bard H , Djian P . Clinical and MRI results in 67 patients operated for gluteus medius and minimus tendon tears with a median follow‐up of 4.6 years. Orthop Traumatol Surg Res. 2014;100(8):849–853. 25453914 10.1016/j.otsr.2014.08.004

[36] Maldonado DR , Annin S , Chen JW , Rosinsky PJ , Shapira J , Lall AC , et al. Full‐thickness gluteus medius tears with or without concomitant hip arthroscopy: minimum 2‐year outcomes using an open approach and contemporary tendon repair techniques. Orthop J Sports Med. 2020;10 8(7):2325967120929330. 10.1177/2325967120929330 PMC7357074 32699803

[37] Maslaris A , Vail TP , Zhang AL , Patel R , Jäger M , Bini SA . Equivalent mid‐term results of open vs endoscopic gluteal tendon tear repair using suture anchors in forty‐five patients. J Arthroplasty. 2020;35(6S):S352–S358. 32279942 10.1016/j.arth.2020.03.013

[38] Maslaris A , Vail TP , Zhang AL , Patel R , Bini SA . Impact of fatty degeneration on the functional outcomes of 38 patients undergoing surgical repair of gluteal tendon tears. Arch Orthop Trauma Surg. 2022;142(9):2173–2183. 33651145 10.1007/s00402-021-03787-2 PMC9381454

[39] Meghpara MB , Bheem R , Haden M , Rosinsky PJ , Shapira J , Maldonado DR , et al. Differences in clinical presentations and surgical outcomes of gluteus medius tears between men and women. Am J Sports Med. 2020;48(14):3594–3602. 33104387 10.1177/0363546520966335

[40] Meghpara MB , Yelton MJ , Annin S , Rosinsky PJ , Shapira J , Maldonado DR , et al. Return to activity after gluteus medius repair in active patients older than 50 years. Orthop J Sports Med. 2021;9(1):2325967120967968. 10.1177/2325967120967968 33553438 PMC7844460

[41] Meghpara MB , Yelton MJ , Annin S , Shapira J , Rosinsky PJ , Maldonado DR , et al. Mid‐term outcomes of endoscopic gluteus medius repair with concomitant arthroscopic labral treatment: a propensity‐matched controlled study. Arthroscopy. 2020;36(11):2856–2865. 32730896 10.1016/j.arthro.2020.07.020

[42] Meghpara MB , Yelton MJ , Glein RM , Malik MS , Rosinsky PJ , Shapira J , et al. Isolated endoscopic gluteus medius repair can achieve successful clinical outcomes at minimum 2‐year follow‐up. Arthrosc Sports Med Rehabil. 2021;3(6):e1697–e1704. 34977622 10.1016/j.asmr.2021.07.026 PMC8689210

[43] Morgan A , Moore M , Derry K , Bi A , Brown J , Youm T , et al. Surgical treatment and outcomes for gluteal tendon tears. Curr Rev Musculoskelet Med. 2024;17(6):157–170. 38619805 10.1007/s12178-024-09896-w PMC11091023

[44] Nadeau NJ , Marder RS , Fasulo SM , Richards SM , Kraeutler MJ , Scillia AJ . Comparable pain levels and functional outcomes with and without the use of dermal allograft augmentation in endoscopic gluteus medius repair. Arthroscopy. 2025;41(6):1799–1805. 39209076 10.1016/j.arthro.2024.08.024

[45] Nazal MR , Abraham PF , Conaway WK , Quinlan NJ , Gillinov SM , Gibbs JS , et al. Endoscopic repair of full‐thickness gluteus medius and minimus tears‐prospective study with a minimum 2‐year follow‐up. Arthroscopy. 2020;36(8):2160–2169. 32387651 10.1016/j.arthro.2020.04.025

[46] Okoroha KR , Beck EC , Nwachukwu BU , Kunze KN , Nho SJ . Defining minimal clinically important difference and patient acceptable symptom state after isolated endoscopic gluteus medius repair. Am J Sports Med. 2019;47(13):3141–3147. 31618066 10.1177/0363546519877179

[47] Page MJ , McKenzie JE , Bossuyt PM , Boutron I , Hoffmann TC , Mulrow CD , et al. The PRISMA 2020 statement: an updated guideline for reporting systematic reviews. BMJ. 2021;372:n71. 33782057 10.1136/bmj.n71 PMC8005924

[48] Paul KD , Hargreaves M , Manfredi JN , Cooke B , Crawford A , Evely T , et al. Patients with operative gluteus medius tears often present with a concomitant history of lumbar pathology. J Orthop. 2024;47:18–22. 38046456 10.1016/j.jor.2023.11.025 PMC10689234

[49] Pearce AN , Stambough JB , Mears SC , Barnes CL , Stronach BM . Diagnosis and treatment options of abductor insufficiency after total hip replacement. Orthop Clin North Am. 2022;53(3):255–265. 35725034 10.1016/j.ocl.2022.03.001

[50] Perets I , Mansor Y , Yuen LC , Chen AW , Chaharbakhshi EO , Domb BG . Endoscopic gluteus medius repair with concomitant arthroscopy for labral tears: a case series with minimum 5‐year outcomes. Arthroscopy. 2017;33(12):2159–2167. 28969951 10.1016/j.arthro.2017.06.032

[51] Prabhavalkar ON , Carbone AD , Curley AJ , Padmanabhan S , Nerys J , Domb BG . Endoscopic tendon compression bridge technique for repair of partial‐thickness gluteus medius tears with concomitant arthroscopy for labral tears: minimum 2‐year outcomes with benchmark control group. Am J Sports Med. 2023;51(14):3764–3771. 37960846 10.1177/03635465231204314

[52] Van Quickenborne D , Van Der Straeten C , Burssens A , Audenaert E . Surgical technique for the open repair of grade 3‐4 tears of the gluteus medius tendon of the hip: technical note and case series. Int J Surg Case Rep. 2025;129:111191. 10.1016/j.ijscr.2025.111191 40132526 PMC11985016

[53] Quinn M , Albright A , Kent V , Morrissey P , Katz L , Kutschke M , et al. Mini‐open technique for gluteus medius tendon repairs is associated with low complication rates and sustained improvement in patient reported outcomes at 2‐year follow‐up. Arthrosc Sports Med Rehabil. 2024;6(5):100972. 10.1016/j.asmr.2024.100972 39534035 PMC11551336

[54] Rajkumar S , Singer GC , Jones JR . Results following repair of gluteus medius defects following total hip arthroplasty. HIP Int. 2011;21(3):293–298. 21698577 10.5301/HIP.2011.8400

[55] Ramadanov N . MCID normalization: a methodological framework for harmonizing heterogeneous PROMs in hip arthroscopy research. J Exp Orthop. 2025;12(4):e70568. 10.1002/jeo2.70568 41245715 PMC12619535

[56] Ramadanov N , Voss M , Diallo RM , Lettner J , Hakam HT , Prill R , et al. Do meta‐analyses of total hip arthroplasty produce reliable results? A systematic review and meta‐epidemiological study of statistical methods. Orthop Surg. 2025;17(7):1936–1955. 40425483 10.1111/os.70077 PMC12214412

[57] Ramadanov N , Voss M , Lott A , Banke IJ . Protocol for systematic review and multilevel meta‐analysis comparing efficacy and safety of endoscopic versus open gluteal tendon repair. BMJ Open. 2025;15(11):e107898. 10.1136/bmjopen-2025-107898 PMC12598952 41213671

[58] Rice MW , Sivasundaram L , Hevesi M , Browning RB , Alter TD , Paul K , et al. Defining the minimal clinically important difference and patient acceptable symptom state after endoscopic gluteus medius or minimus repair with or without labral treatment and routine capsular closure at minimum 5‐year follow‐up. Am J Sports Med. 2022;50(10):2629–2636. 35913620 10.1177/03635465221105469

[59] Rice MW , Browning RB , Fenn TW , Hevesi M , Nho SJ . Defining the minimal clinically important difference (MCID) and patient acceptable symptom state (PASS) at 2 years following open gluteus medius and/or minimus repair. J Hip Preserv Surg. 2023;11(2):92–97. 39070207 10.1093/jhps/hnad019 PMC11272634

[60] Rosinsky PJ , Bheem R , Meghpara MB , Haden M , Shapira J , Maldonado DR , et al. Asymptomatic gluteal tendinopathies negatively impact outcomes of total hip arthroplasty: a propensity score‐matched study. J Arthroplasty. 2021;36(1):242–249. 32828621 10.1016/j.arth.2020.07.063

[61] Saltzman BM , Ukwuani G , Makhni EC , Stephens JP , Nho SJ . The effect of platelet‐rich fibrin matrix at the time of gluteus medius repair: a retrospective comparative study. Arthroscopy. 2018;34(3):832–841. 29287951 10.1016/j.arthro.2017.09.032

[62] Schröder JH , Geßlein M , Schütz M , Perka C , Krüger D . Offene Refixation von Gluteus medius und minimus in Double‐Row‐Technik: Klinische und radiologische Ergebnisse. Orthopade. 2018;47(3):238–245. [German]. 29327070 10.1007/s00132-017-3524-1

[63] Shea BJ , Reeves BC , Wells G , Thuku M , Hamel C , Moran J , et al. AMSTAR 2: a critical appraisal tool for systematic reviews that include randomised or non‐randomised studies of healthcare interventions, or both. BMJ. 2017;358:j4008. 28935701 10.1136/bmj.j4008 PMC5833365

[64] Sterne JAC , Savović J , Page MJ , Elbers RG , Blencowe NS , Boutron I , et al. RoB 2: a revised tool for assessing risk of bias in randomised trials. BMJ. 2019;366:l4898. 31462531 10.1136/bmj.l4898

[65] Sterne JA , Hernán MA , Reeves BC , Savović J , Berkman ND , Viswanathan M , et al. ROBINS‐I: a tool for assessing risk of bias in non‐randomised studies of interventions. BMJ. 2016;355:i4919. 27733354 10.1136/bmj.i4919 PMC5062054

[66] Sterne JAC , Egger M . Funnel plots for detecting bias in meta‐analysis. J Clin Epidemiol. 2001;54(10):1046–1055. 11576817 10.1016/s0895-4356(01)00377-8

[67] Thaunat M , Chatellard R , Noël E , Sonnery‐Cottet B , Nové‐Josserand L . Endoscopic repair of partial‐thickness undersurface tears of the gluteus medius tendon. Orthop Traumatol Surg Res. 2013;99(7):853–857. 24075011 10.1016/j.otsr.2013.06.005

[68] Thaunat M , Clowez G , Desseaux A , Murphy CG , Sbiyaa M , Noël E , et al. Influence of muscle fatty degeneration on functional outcomes after endoscopic gluteus medius repair. Arthroscopy. 2018;34(6):1816–1824. 29573934 10.1016/j.arthro.2018.01.005

[69] Thaunat M , de Saint Vincent B , Caron E , Ingale PS . A comparison of outcomes after endoscopic repair of partial‐ versus full‐thickness tears of the gluteus medius tendon. Arthroscopy. 2021;37(8):2465–2472. 33631252 10.1016/j.arthro.2021.02.020

[70] Twardy V , Warnecke D , Prodinger PM , Harrasser N , Scheele C , von Eisenhart‐Rothe R , et al. Superior primary stability of a knotless double‐row construct compared to Mason‐Allen repair for anatomical refixation of gluteal tendons‐biomechanical human cadaver study. Orthop Surg. 2025;17(10):2973–2981. 40803746 10.1111/os.70153 PMC12497568

[71] Vogel MJ , Jan K , Alvero AB , Gilat R , Ebersole JW , Nho SJ . Hip gluteus repair yields satisfactory clinically significant outcome achievement by 1 year in a majority of patients: those with partial‐thickness tears and preoperative hip abduction weakness show delayed recovery. Arthroscopy. 2024;40(12):2852–2858. 38508287 10.1016/j.arthro.2024.02.044

[72] Voos JE , Shindle MK , Pruett A , Asnis PD , Kelly BT . Endoscopic repair of gluteus medius tendon tears of the hip. Am J Sports Med. 2009;37(4):743–747. 19204363 10.1177/0363546508328412

[73] Walsh MJ , Walton JR , Walsh NA . Surgical repair of the gluteal tendons. J Arthroplasty. 2011;26(8):1514–1519. 21798694 10.1016/j.arth.2011.03.004

[74] Weir CJ , Butcher I , Assi V , Lewis SC , Murray GD , Langhorne P , et al. Dealing with missing standard deviation and mean values in meta‐analysis of continuous outcomes: a systematic review. BMC Med Res Methodol 2018;18(1):25. 29514597 10.1186/s12874-018-0483-0 PMC5842611

