# Supplementary figures and images for: Endoscopic gluteal tendon repair reduces complication rates while achieving outcomes comparable to open repair: A multilevel meta‐analysis
Source: Knee Surg Sports Traumatol Arthrosc. 2026 Jan 31;34(3):1061–80. doi: 10.1002/ksa.70309 (PMC12948349; doi:10.1002/ksa.70309)

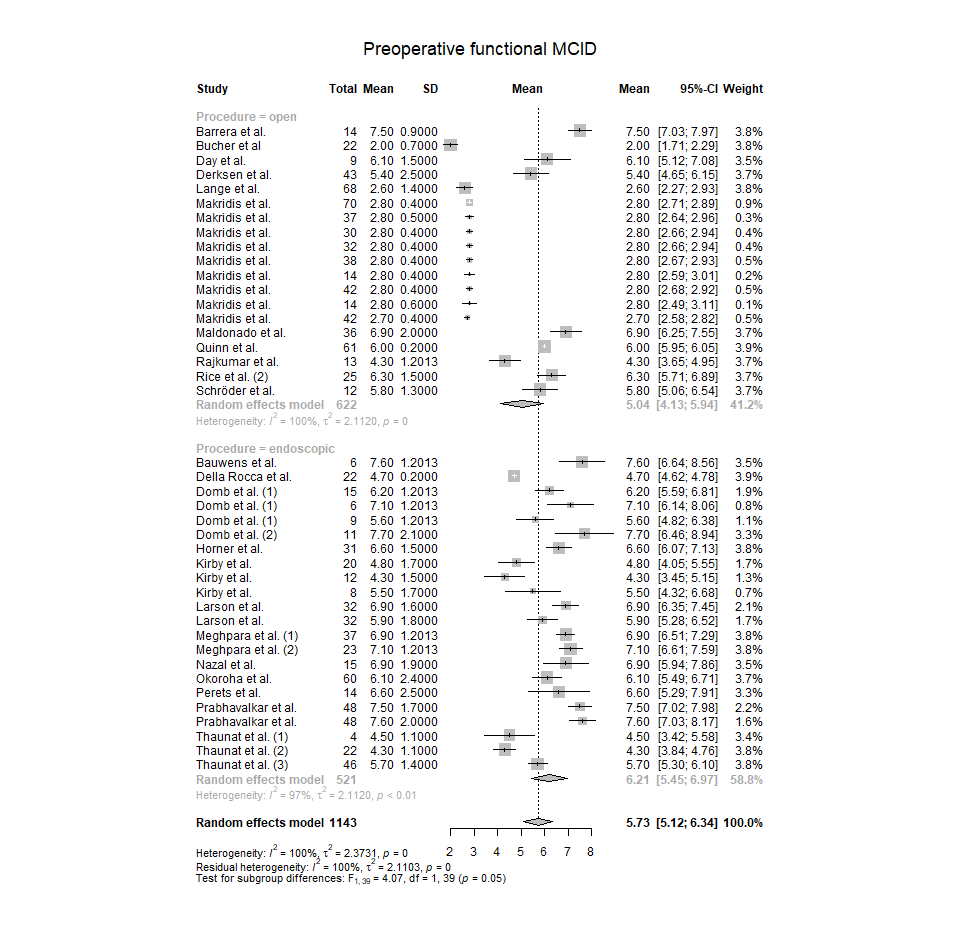

Supplement: Supplementary file 1 — Supporting information. [file KSA-34-1061-s009.png]

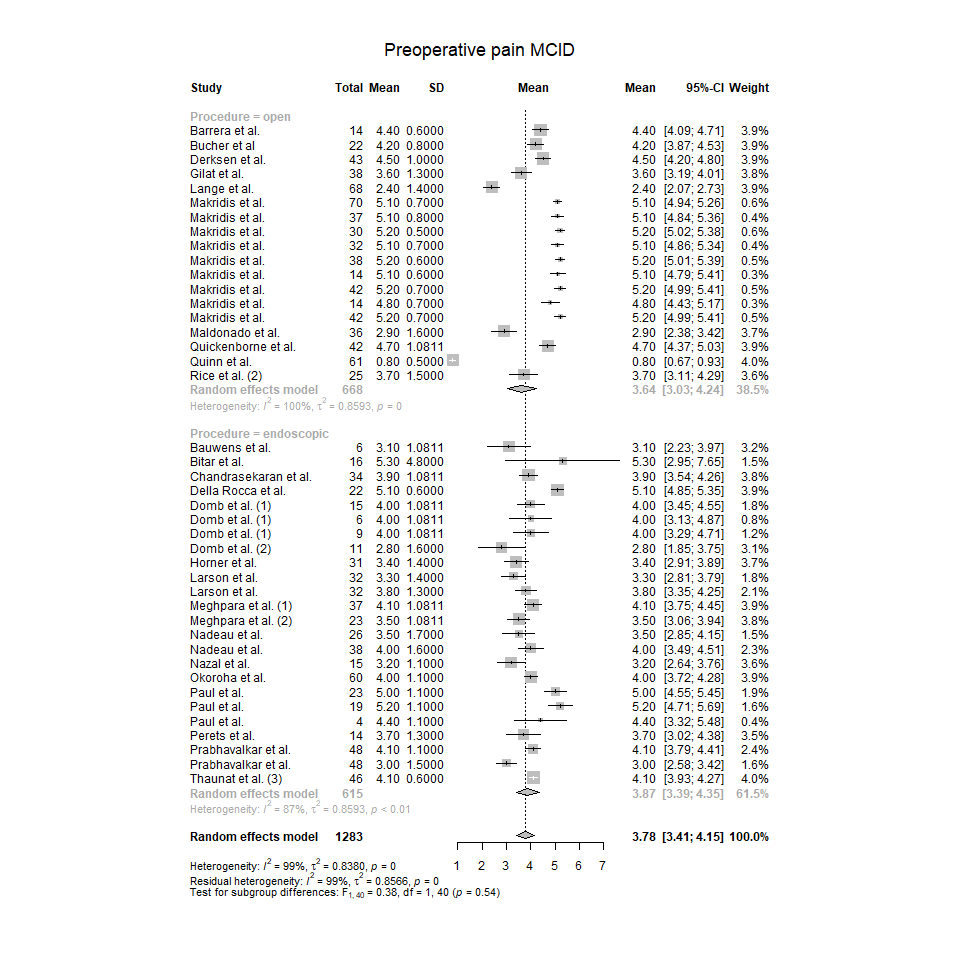

Supplement: Supplementary file 2 — Supporting information. [file KSA-34-1061-s013.png]

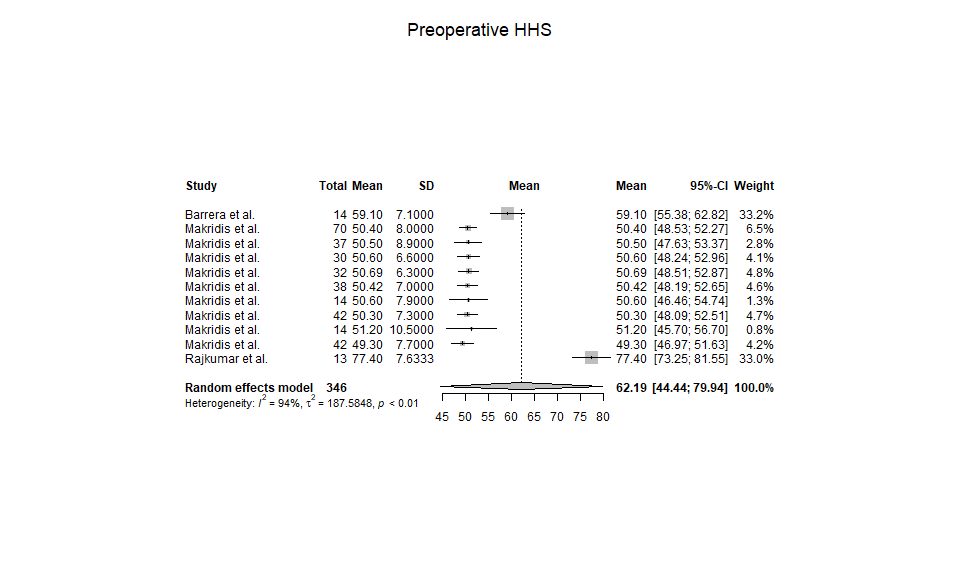

Supplement: Supplementary file 3 — Supporting information. [file KSA-34-1061-s021.png]

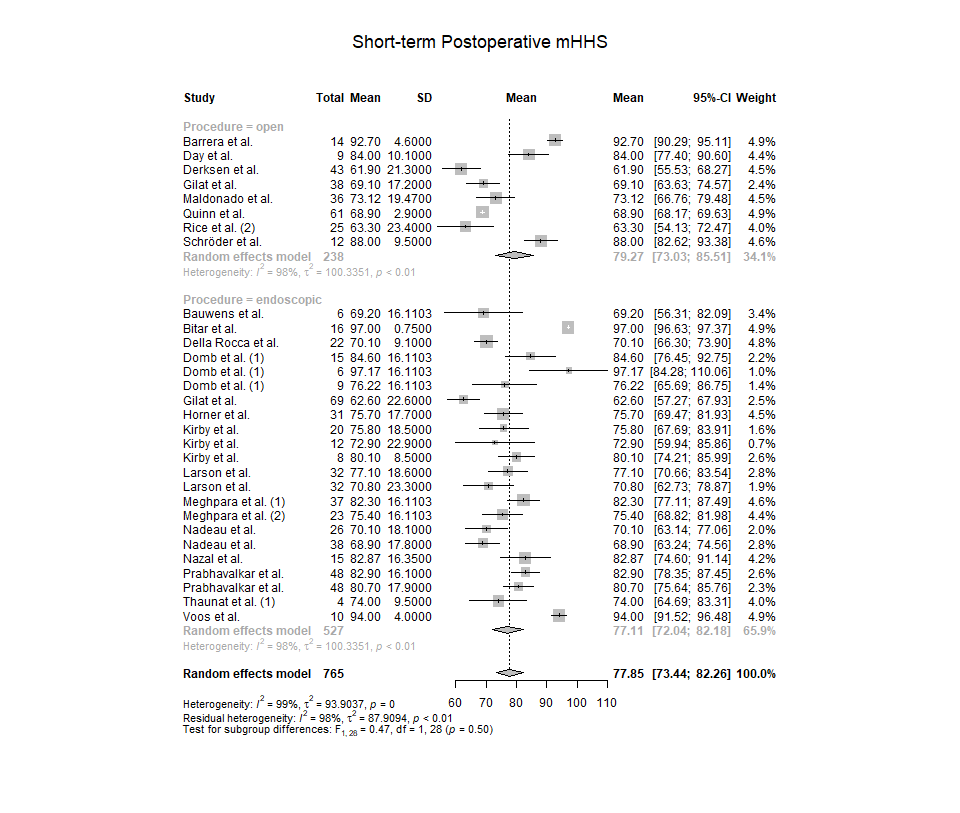

Supplement: Supplementary file 4 — Supporting information. [file KSA-34-1061-s008.png]

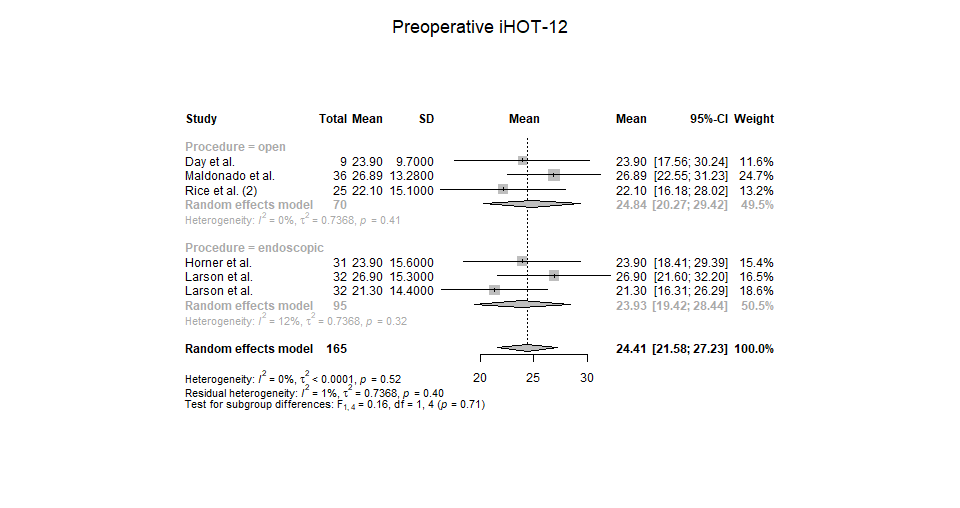

Supplement: Supplementary file 5 — Supporting information. [file KSA-34-1061-s024.png]

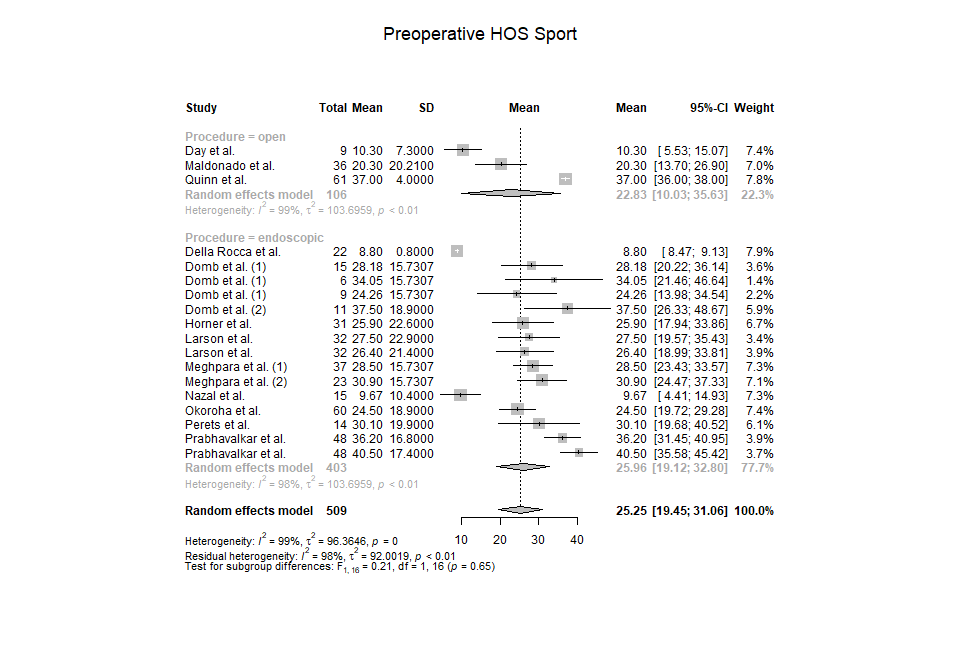

Supplement: Supplementary file 6 — Supporting information. [file KSA-34-1061-s026.png]

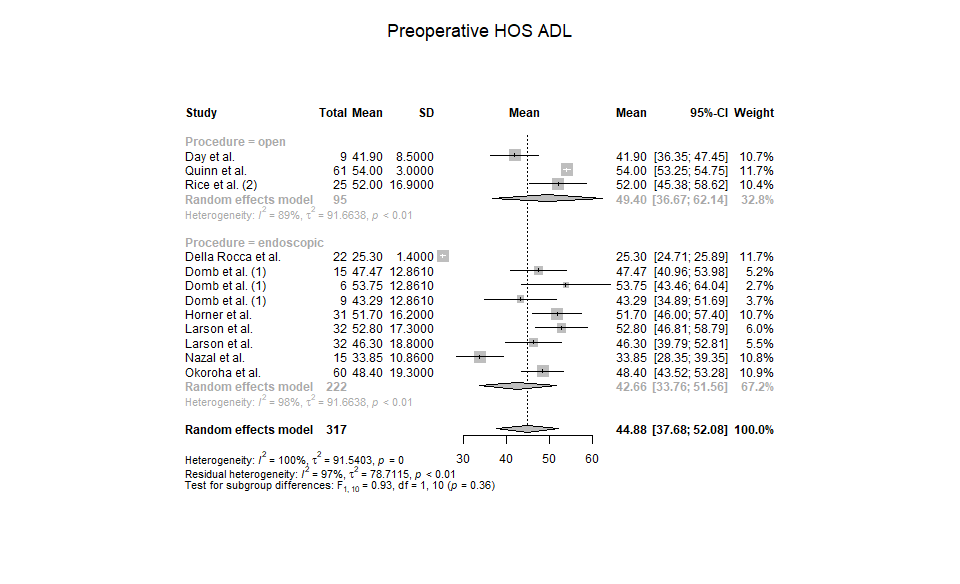

Supplement: Supplementary file 7 — Supporting information. [file KSA-34-1061-s004.png]

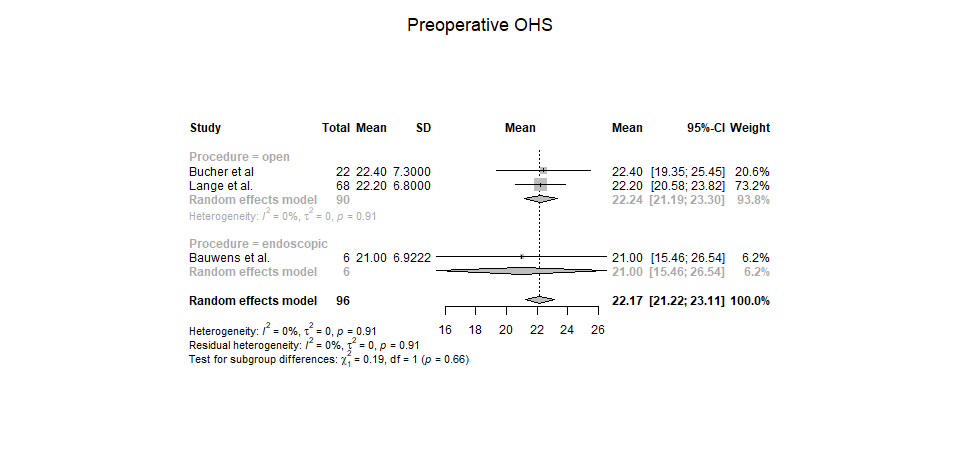

Supplement: Supplementary file 8 — Supporting information. [file KSA-34-1061-s015.png]

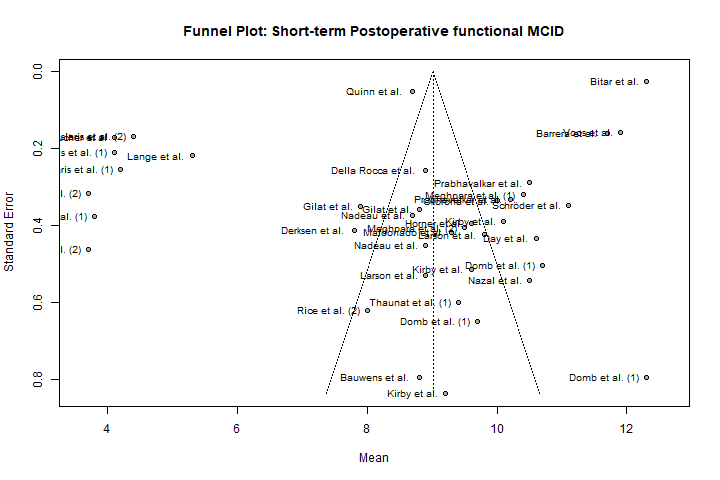

Supplement: Supplementary file 9 — Supporting information. [file KSA-34-1061-s035.png]

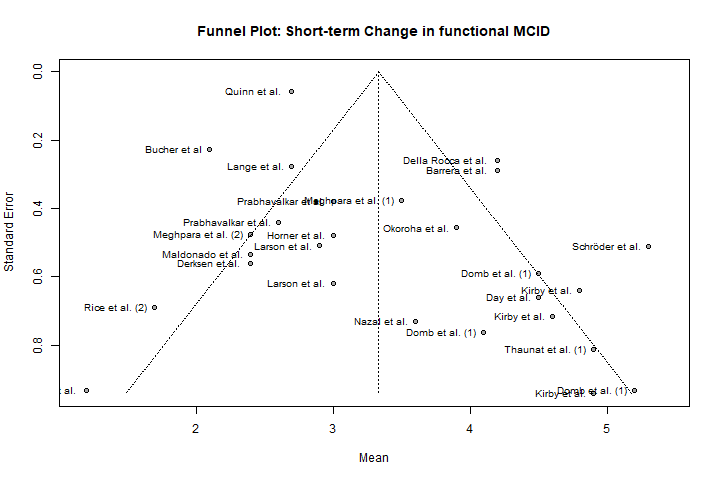

Supplement: Supplementary file 10 — Supporting information. [file KSA-34-1061-s036.png]

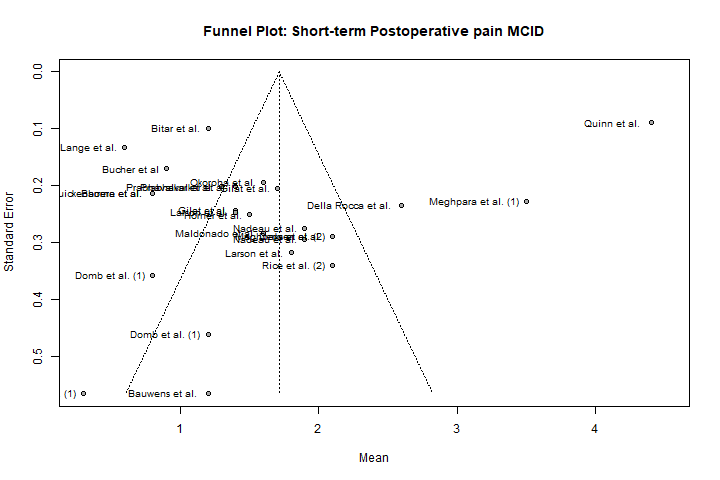

Supplement: Supplementary file 11 — Supporting information. [file KSA-34-1061-s012.png]

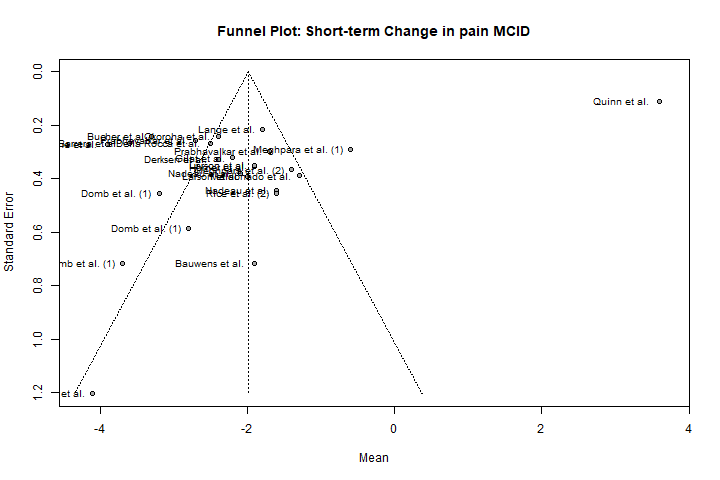

Supplement: Supplementary file 12 — Supporting information. [file KSA-34-1061-s020.png]

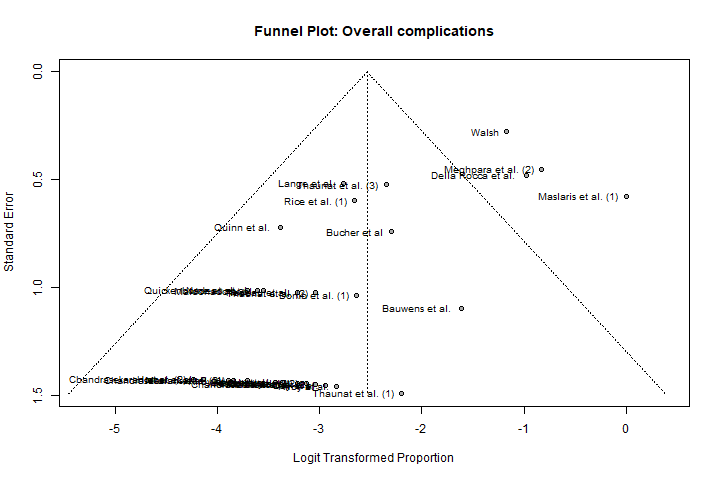

Supplement: Supplementary file 13 — Supporting information. [file KSA-34-1061-s016.png]

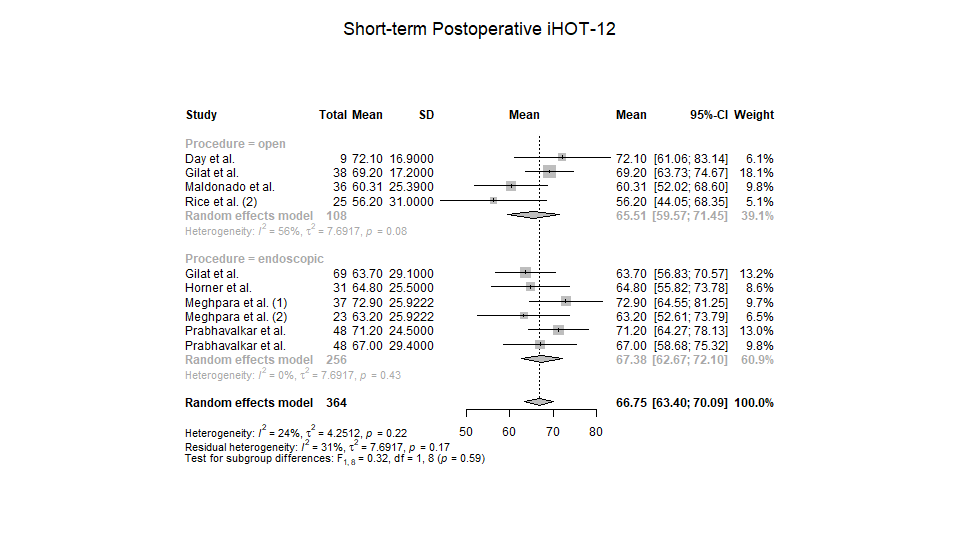

Supplement: Supplementary file 14 — Supporting information. [file KSA-34-1061-s002.png]

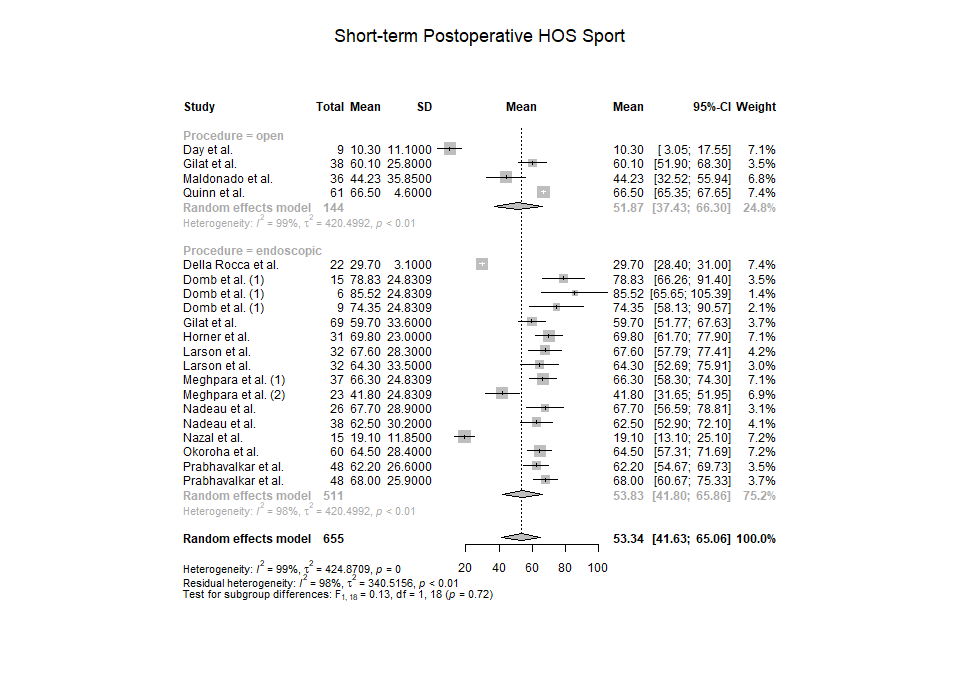

Supplement: Supplementary file 15 — Supporting information. [file KSA-34-1061-s030.png]

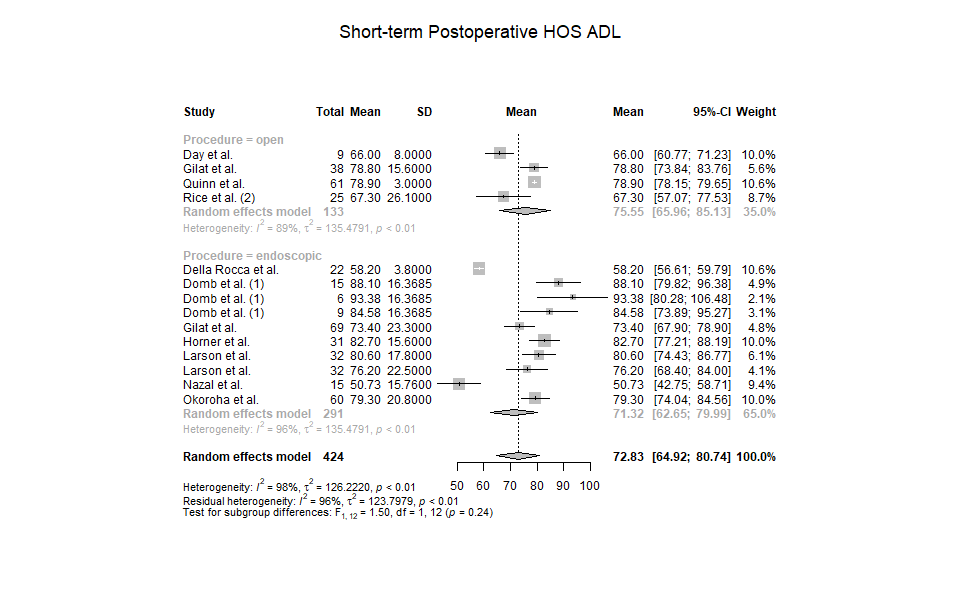

Supplement: Supplementary file 16 — Supporting information. [file KSA-34-1061-s019.png]

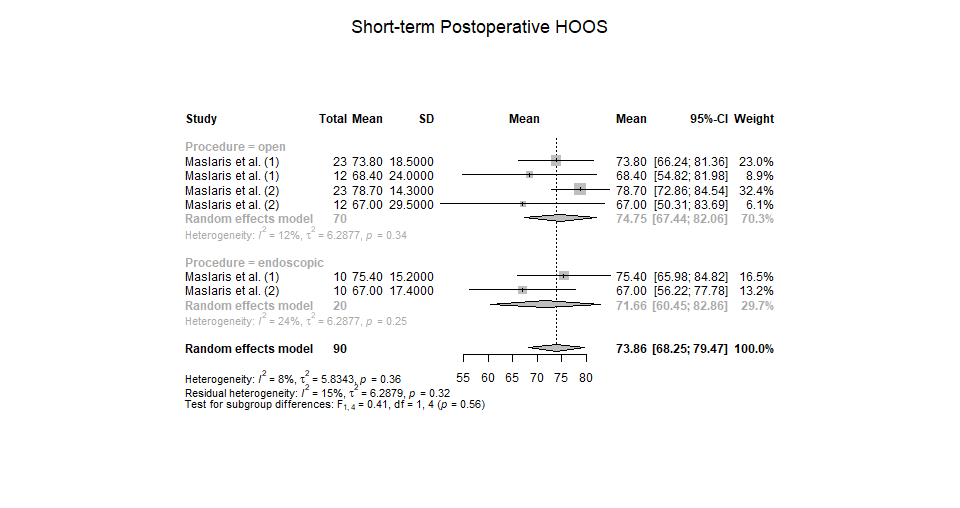

Supplement: Supplementary file 17 — Supporting information. [file KSA-34-1061-s025.png]

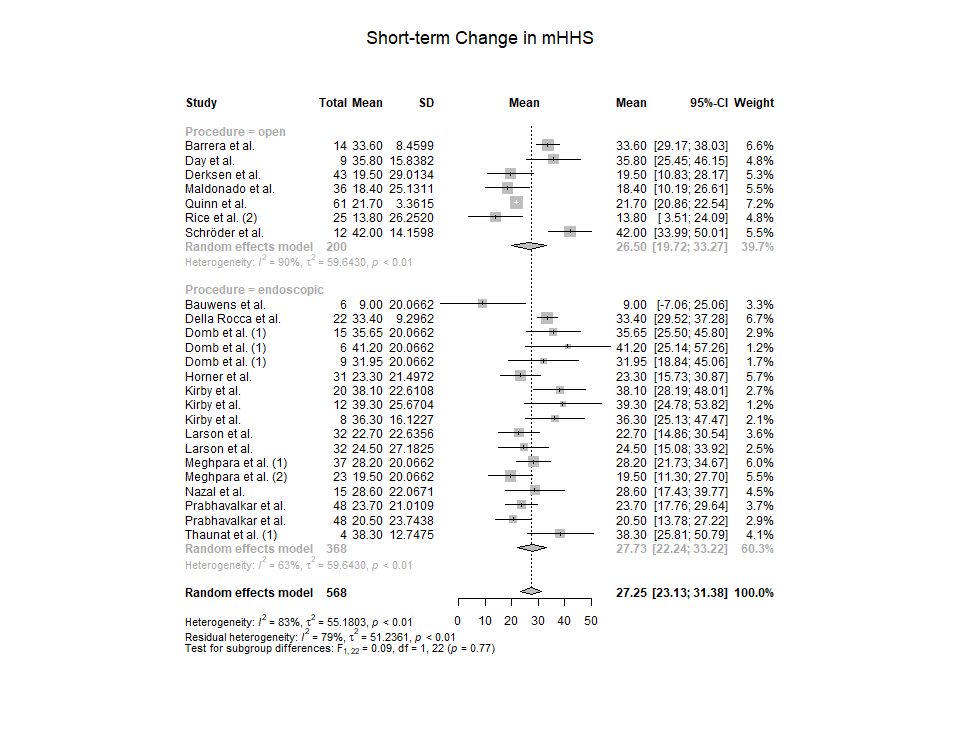

Supplement: Supplementary file 18 — Supporting information. [file KSA-34-1061-s033.png]

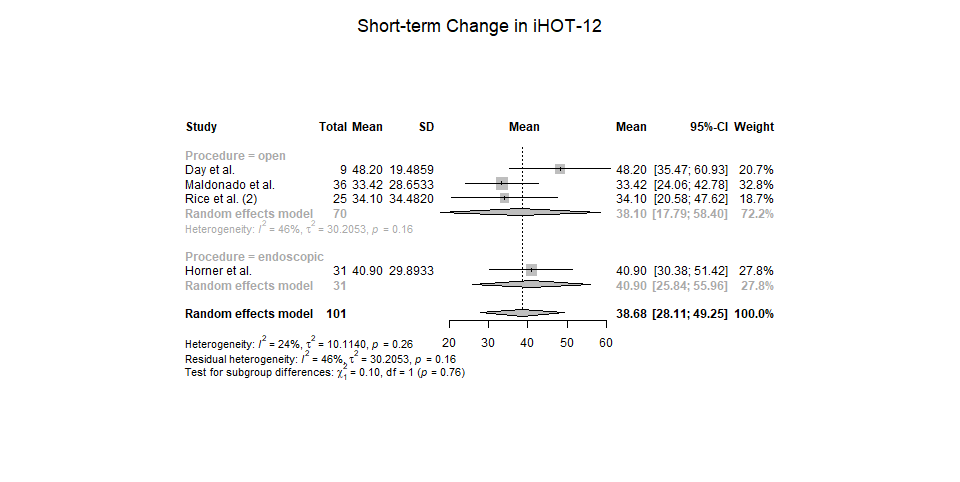

Supplement: Supplementary file 19 — Supporting information. [file KSA-34-1061-s005.png]

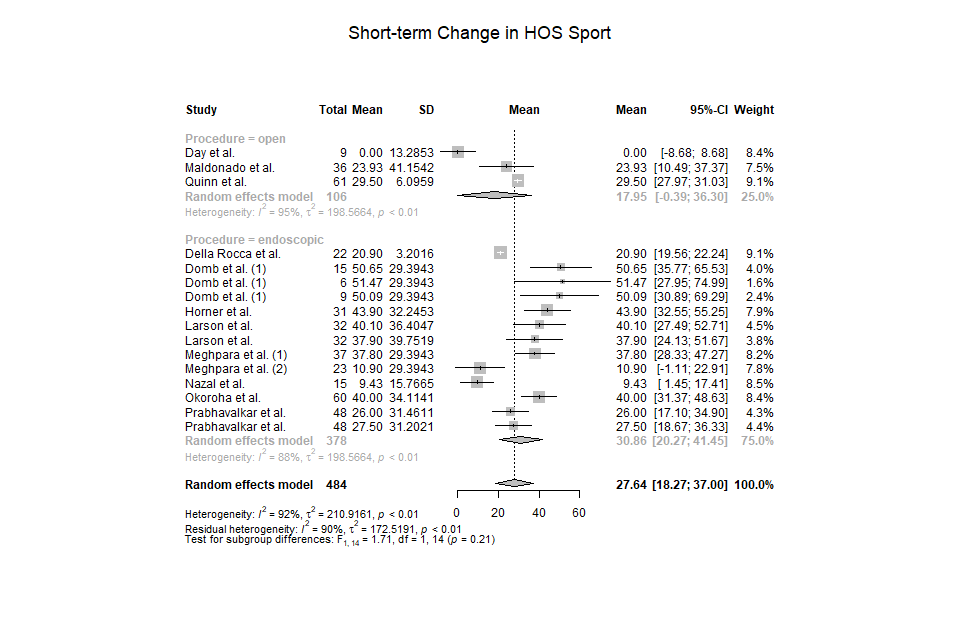

Supplement: Supplementary file 20 — Supporting information. [file KSA-34-1061-s032.png]

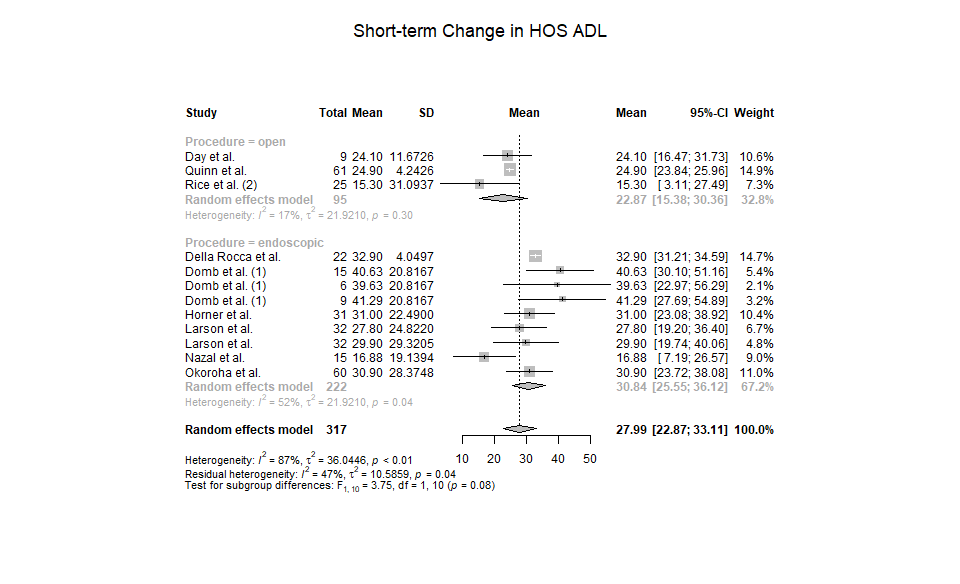

Supplement: Supplementary file 21 — Supporting information. [file KSA-34-1061-s006.png]

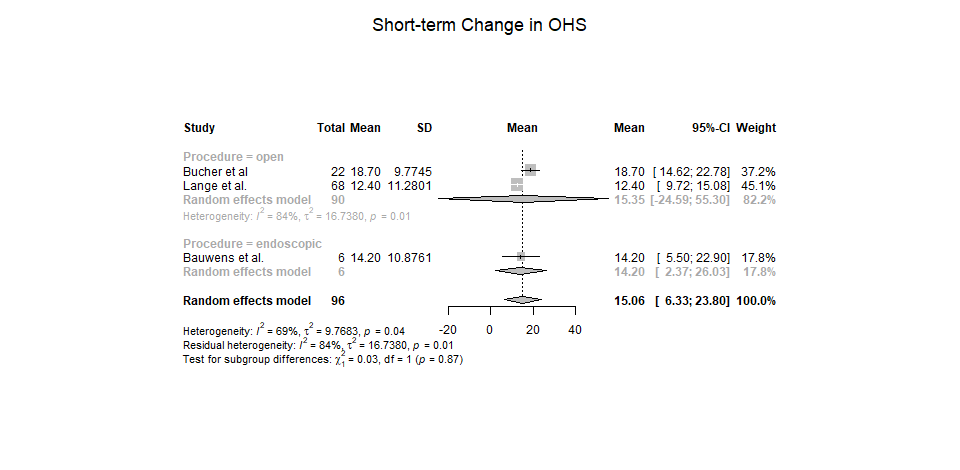

Supplement: Supplementary file 22 — Supporting information. [file KSA-34-1061-s023.png]

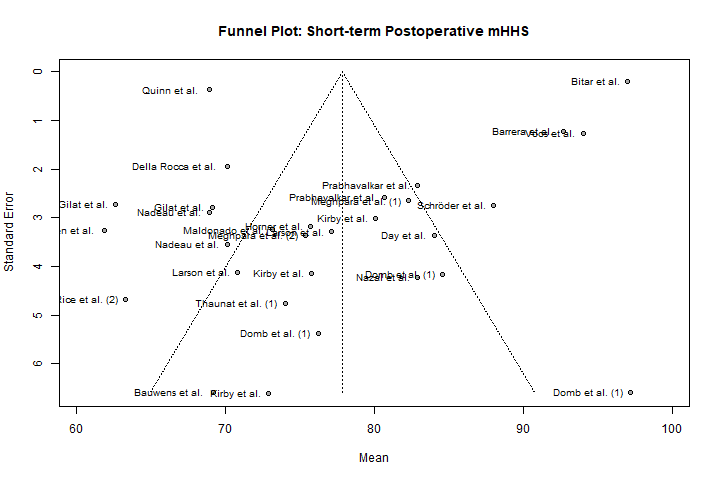

Supplement: Supplementary file 23 — Supporting information. [file KSA-34-1061-s018.png]

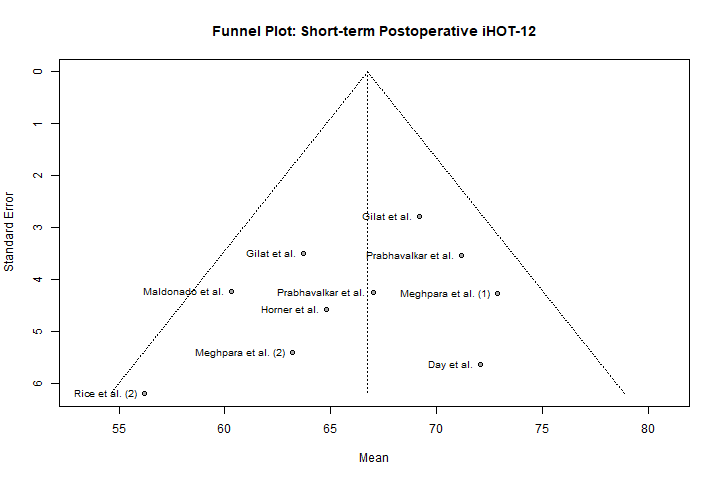

Supplement: Supplementary file 24 — Supporting information. [file KSA-34-1061-s010.png]

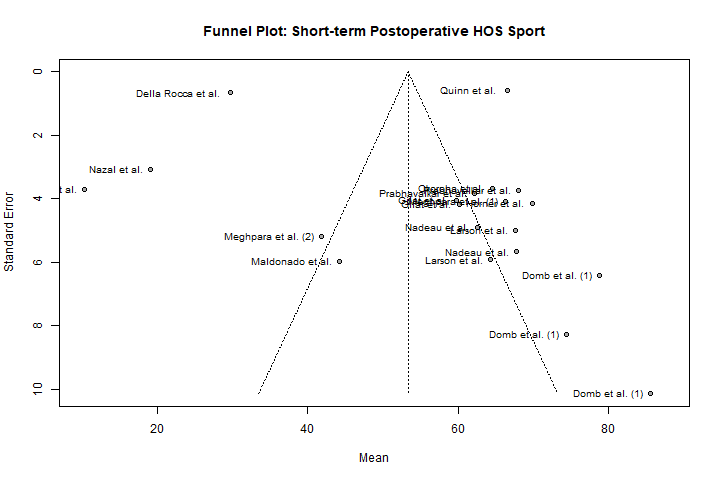

Supplement: Supplementary file 25 — Supporting information. [file KSA-34-1061-s034.png]

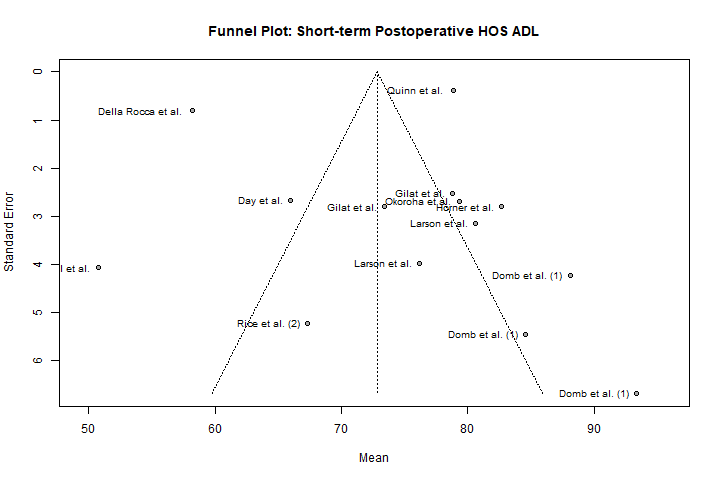

Supplement: Supplementary file 26 — Supporting information. [file KSA-34-1061-s017.png]

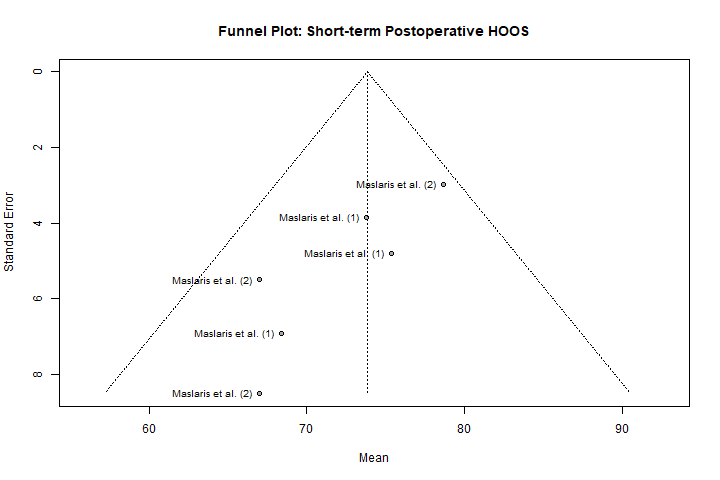

Supplement: Supplementary file 27 — Supporting information. [file KSA-34-1061-s022.png]

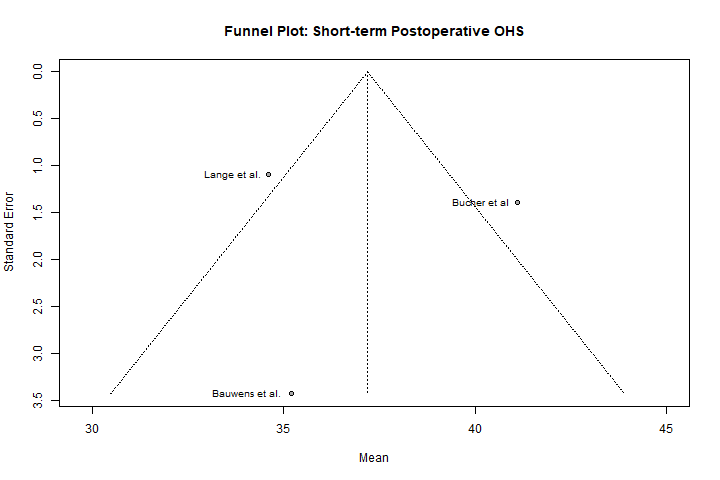

Supplement: Supplementary file 28 — Supporting information. [file KSA-34-1061-s037.png]

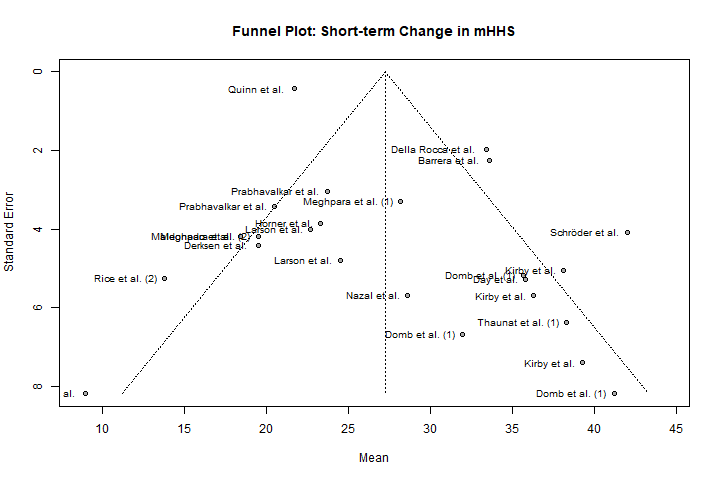

Supplement: Supplementary file 29 — Supporting information. [file KSA-34-1061-s011.png]

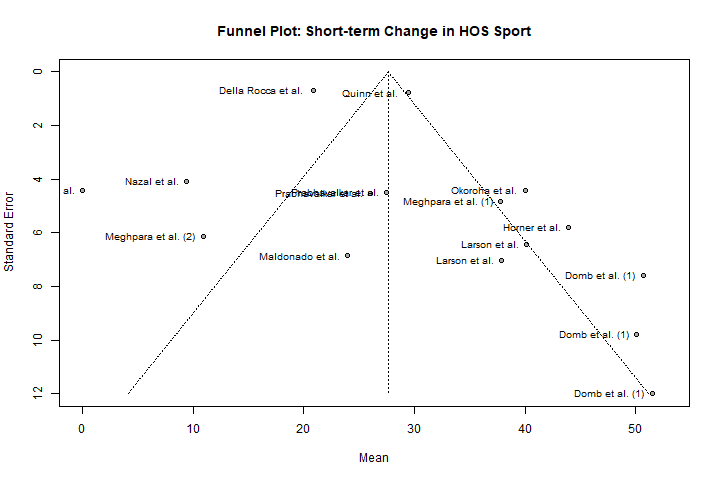

Supplement: Supplementary file 30 — Supporting information. [file KSA-34-1061-s029.png]

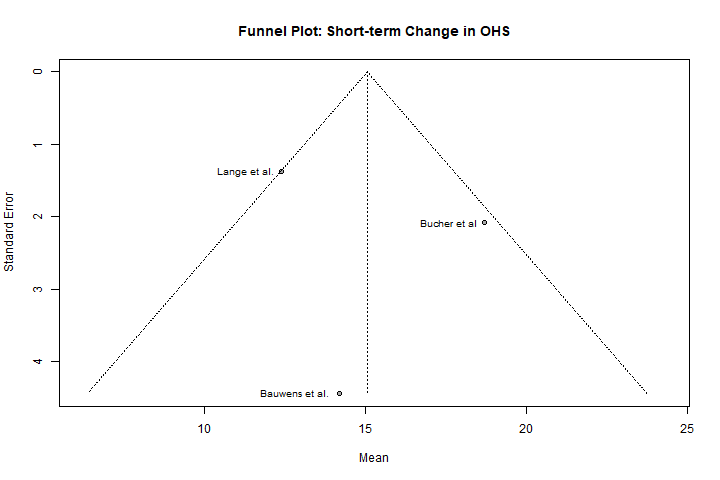

Supplement: Supplementary file 31 — Supporting information. [file KSA-34-1061-s007.png]

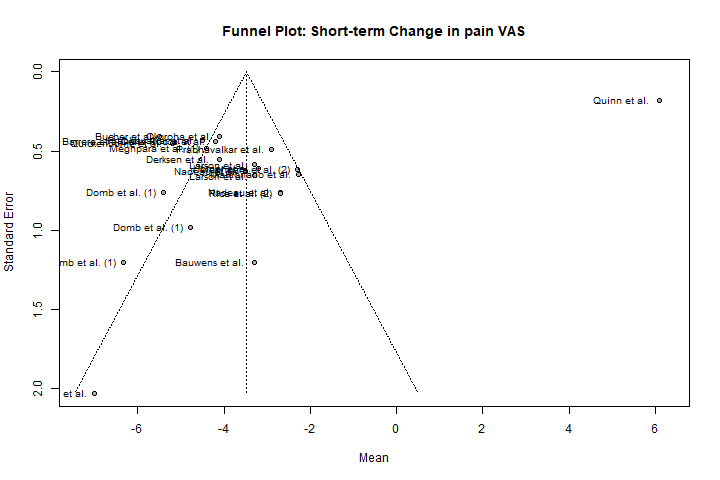

Supplement: Supplementary file 32 — Supporting information. [file KSA-34-1061-s028.png]

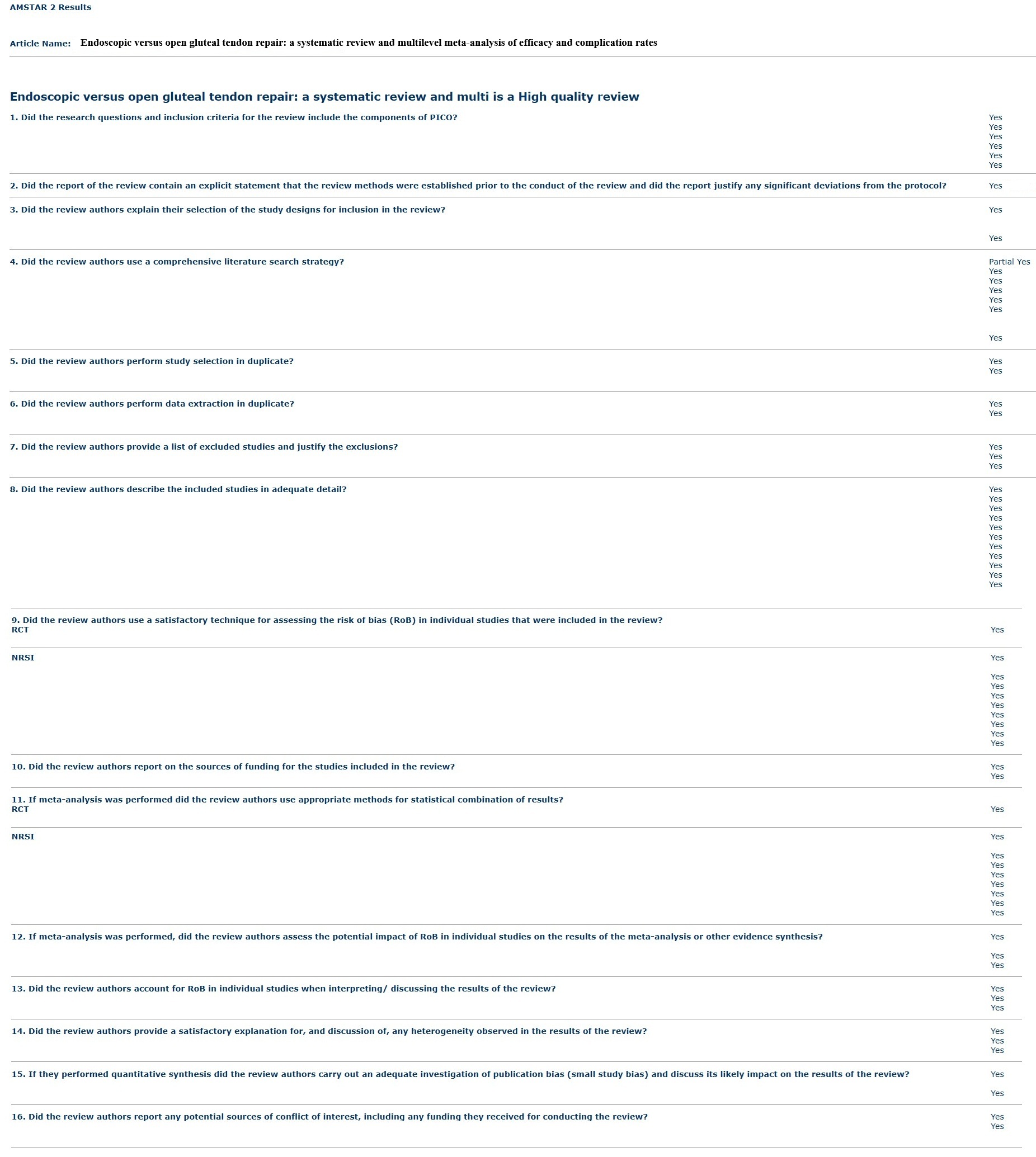

Supplement: Supplementary file 35 — Supporting information. [file KSA-34-1061-s001.jpg]
