# Supplementary material for: Endoscopic gluteal tendon repair reduces complication rates while achieving outcomes comparable to open repair: A multilevel meta‐analysis
Source: Knee Surg Sports Traumatol Arthrosc. 2026 Jan 31;34(3):1061–80. doi: 10.1002/ksa.70309 (PMC12948349; doi:10.1002/ksa.70309)
Supplement: Supplementary file 36 — Supporting information. [file KSA-34-1061-s003.docx]

|  | Operative procedure | Primary studies, N | Hips, N | Mean value | Confidence interval | τ^2^ | I^2^ | Heterogeneity: p | Difference: p |
| --- | --- | --- | --- | --- | --- | --- | --- | --- | --- |
| Preoperative functional MCID | Total | 41 | 1143 | 5.73 | 5.12 ; 6.34 | 2,37 | 1 | < 0.0001 *** | 0.0507 |
|  | Open | 19 | 622 | 5.04 | 4.13 ; 5.94 | 2,11 | 1 | < 0.0001 *** |  |
|  | Endoscopic | 22 | 521 | 6.21 | 5.45 ; 6.97 | 2,11 | 0,97 | < 0.0001 *** |  |
| Postoperative functional MCID | Total | 39 | 1005 | 9.01 | 8.11 ; 9.91 | 5,23 | 1 | < 0.0001 *** | 0.6265 |
|  | Open | 14 | 398 | 9.09 | 8.12 ; 10.06 | 5,36 | 0,99 | < 0.0001 *** |  |
|  | Endoscopic | 25 | 607 | 8.96 | 8.02 ; 9.9 | 5,36 | 0,99 | < 0.0001 *** |  |
| Change in functional MCID | Total | 27 | 718 | 3.33 | 2.86 ; 3.81 | 0,87 | 0,83 | < 0.0001 *** | 0.3635 |
|  | Open | 9 | 290 | 3.10 | 2.39 ; 3.81 | 0,87 | 0,88 | < 0.0001 *** |  |
|  | Endoscopic | 18 | 428 | 3.52 | 2.89 ; 4.15 | 0,87 | 0,63 | 0.0001 *** |  |
| Preoperative pain MCID | Total | 42 | 1283 | 3.78 | 3.41 ; 4.15 | 0,84 | 0,99 | < 0.0001 *** | 0.5414 |
|  | Open | 18 | 668 | 3.64 | 3.03 ; 4.24 | 0,86 | 1 | < 0.0001 *** |  |
|  | Endoscopic | 24 | 615 | 3.87 | 3.39 ; 4.35 | 0,86 | 0,87 | < 0.0001 *** |  |
| Postoperative pain MCID | Total | 26 | 867 | 1.71 | 1.27 ; 2.16 | 0,88 | 0,98 | < 0.0001 *** | 0.3787 |
|  | Open | 9 | 349 | 1.58 | 1.03 ; 2.13 | 0,9 | 0,99 | < 0.0001 *** |  |
|  | Endoscopic | 17 | 518 | 1.81 | 1.31 ; 2.32 | 0,9 | 0,87 | < 0.0001 *** |  |
| Change in pain MCID | Total | 25 | 798 | -1.99 | -2.74 ; -1.24 | 2,5 | 0,99 | < 0.0001 *** | 0.6822 |
|  | Open | 9 | 349 | -1.82 | -2.96 ; -0.69 | 2,62 | 1 | < 0.0001 *** |  |
|  | Endoscopic | 16 | 449 | -2.13 | -3.18 ; -1.09 | 2,62 | 0,74 | < 0.0001 *** |  |
| Complications | Total | 32 | 804 | 0.07 | 0.05 ; 0.11 | 0,77 | 0,53 | 0.0002 *** | 0.6953 |
|  | Open | 11 | 371 | 0.08 | 0.04 ; 0.16 | 0,81 | 0,71 | 0.0001 *** |  |
|  | Endoscopic | 21 | 433 | 0.07 | 0.04 ; 0.12 | 0,81 | 0,33 | 0.0746 |  |
| Preoperative mHHS | Total | 28 | 661 | 49.07 | 45.49 ; 52.64 | 59,91 | 0,97 | < 0.0001 *** | 0.816 |
|  | Open | 7 | 200 | 49.69 | 43.2 ; 56.18 | 62,8 | 0,88 | < 0.0001 *** |  |
|  | Endoscopic | 21 | 461 | 48.79 | 44.36 ; 53.22 | 62,8 | 0,96 | < 0.0001 *** |  |
| Postoperative mHHS | Total | 30 | 765 | 77.85 | 73.44 ; 82.26 | 93,9 | 0,99 | < 0.0001 *** | 0.5005 |
|  | Open | 8 | 238 | 79.27 | 73.03 ; 85.51 | 100,34 | 0,98 | < 0.0001 *** |  |
|  | Endoscopic | 22 | 527 | 77.11 | 72.04 ; 82.18 | 100,34 | 0,98 | < 0.0001 *** |  |
| Change in mHHS | Total | 24 | 568 | 27.25 | 23.13 ; 31.38 | 55,18 | 0,83 | < 0.0001 *** | 0.7722 |
|  | Open | 7 | 200 | 26.5 | 19.72 ; 33.27 | 59,64 | 0,9 | < 0.0001 *** |  |
|  | Endoscopic | 17 | 368 | 27.73 | 22.24 ; 33.22 | 59,64 | 0,63 | 0.0002 *** |  |
| Preoperative iHOT-12 | Total | 6 | 165 | 24.41 | 21.58 ; 27.23 | 0 | 0 | 0.5156 | 0.7134 |
|  | Open | 3 | 70 | 24.84 | 20.27 ; 29.42 | 0,74 | 0 | 0.414 |  |
|  | Endoscopic | 3 | 95 | 23.93 | 19.42 ; 28.44 | 0,74 | 0,12 | 0.3209 |  |
| Postoperative iHOT-12 | Total | 10 | 364 | 66.75 | 63.4 ; 70.09 | 4,25 | 0,24 | 0.2184 | 0.5858 |
|  | Open | 4 | 108 | 65.51 | 59.57 ; 71.45 | 7,69 | 0,56 | 0.0789 |  |
|  | Endoscopic | 6 | 256 | 67.38 | 62.67 ; 72.1 | 7,69 | 0 | 0.4319 |  |
| Change in iHOT-12 | Total | 4 | 101 | 38.68 | 28.11 ; 49.25 | 10,11 | 0,24 | 0.2649 | 0.7558 |
|  | Open | 3 | 70 | 38.1 | 17.79 ; 58.4 | 30,21 | 0,46 | 0.1583 |  |
|  | Endoscopic | 1 | 31 | 40.9 | 25.84 ; 55.96 | 30,21 |  | - |  |
| Preoperative HOS-ADL | Total | 12 | 317 | 44.88 | 37.68 ; 52.08 | 91,54 | 1 | < 0.0001 *** | 0.3564 |
|  | Open | 3 | 95 | 49.4 | 36.67 ; 62.14 | 91,66 | 0,89 | 0.0001 *** |  |
|  | Endoscopic | 9 | 222 | 42.66 | 33.76 ; 51.56 | 91,66 | 0,98 | < 0.0001 *** |  |
| Postoperative HOS-ADL | Total | 14 | 424 | 72.83 | 64.92 ; 80.74 | 126,22 | 0,98 | < 0.0001 *** | 0.2435 |
|  | Open | 4 | 133 | 75.55 | 65.96 ; 85.13 | 135,48 | 0,89 | < 0.0001 *** |  |
|  | Endoscopic | 10 | 291 | 71.32 | 62.65 ; 79.99 | 135,48 | 0,96 | < 0.0001 *** |  |
| Change in HOS-ADL | Total | 12 | 317 | 27.99 | 22.87 ; 33.11 | 36,04 | 0,87 | < 0.0001 *** | 0.0814 |
|  | Open | 3 | 95 | 22.87 | 15.38 ; 30.36 | 21,92 | 0,17 | 0.3013 |  |
|  | Endoscopic | 9 | 222 | 30.84 | 25.55 ; 36.12 | 21,92 | 0,52 | 0.0355 * |  |
| Preoperative HOS-SSS | Total | 18 | 509 | 25.25 | 19.45 ; 31.06 | 96,36 | 0,99 | < 0.0001 *** | 0.6538 |
|  | Open | 3 | 106 | 22.83 | 10.03 ; 35.63 | 103,7 | 0,99 | < 0.0001 *** |  |
|  | Endoscopic | 15 | 403 | 25.96 | 19.12 ; 32.8 | 103,7 | 0,98 | < 0.0001 *** |  |
| Postoperative HOS-SSS | Total | 20 | 655 | 53.34 | 41.63 ; 65.06 | 424,87 | 0,99 | < 0.0001 *** | 0.7177 |
|  | Open | 4 | 144 | 51.87 | 37.43 ; 66.3 | 420,5 | 0,99 | < 0.0001 *** |  |
|  | Endoscopic | 16 | 511 | 53.83 | 41.8 ; 65.86 | 420,5 | 0,98 | < 0.0001 *** |  |
| Change in HOS-SSS | Total | 16 | 484 | 27.64 | 18.27 ; 37 | 210,92 | 0,92 | < 0.0001 *** | 0.2124 |
|  | Open | 3 | 106 | 17.95 | -0.39 ; 36.3 | 198,57 | 0,95 | < 0.0001 *** |  |
|  | Endoscopic | 13 | 378 | 30.86 | 20.27 ; 41.45 | 198,57 | 0,88 | < 0.0001 *** |  |
| Preoperative OHS | Total | 3 | 96 | 22.17 | 21.22 ; 23.11 | 0 | 0 | 0.9073 | 0.66 |
|  | Open | 2 | 90 | 22.24 | 21.19 ; 23.3 | 0 | 0 | 0.9096 |  |
|  | Endoscopic | 1 | 6 | 21.00 | 15.46 ; 26.54 | 0 |  | - |  |
| Postperative OHS | Total | 3 | 96 | 37.19 | 27.89 ; 46.49 | 12,16 | 0,86 | 0.0010 *** | 0.6885 |
|  | Open | 2 | 90 | 37.79 | -3.5 ; 79.08 | 19,57 | 0,93 | 0.0002 *** |  |
|  | Endoscopic | 1 | 6 | 35.20 | 24.24 ; 46.16 | 19,57 |  | - |  |
| Change in OHS | Total | 3 | 96 | 15.06 | 6.33 ; 23.8 | 9,77 | 0,69 | 0.0410 * | 0.8654 |
|  | Open | 2 | 90 | 15.35 | -24.59 ; 55.3 | 16,74 | 0,84 | 0.0115 * |  |
|  | Endoscopic | 1 | 6 | 14.20 | 2.37 ; 26.03 | 16,74 |  | - |  |
| Postoperative HOOS | Total | 6 | 90 | 73.86 | 68.25 ; 79.47 | 5,83 | 0,08 | 0.3623 | 0.5559 |
|  | Open | 4 | 70 | 74.75 | 67.44 ; 82.06 | 6,29 | 0,12 | 0.3352 |  |
|  | Endoscopic | 2 | 20 | 71.66 | 60.45 ; 82.86 | 6,29 | 0,24 | 0.2503 |  |
| Preoperative pain VAS | Total | 40 | 1177 | 6.51 | 5.86 ; 7.16 | 2,38 | 0,99 | < 0.0001 *** | 0.8592 |
|  | Open | 16 | 562 | 6.43 | 5.28 ; 7.58 | 2,49 | 1 | < 0.0001 *** |  |
|  | Endoscopic | 24 | 615 | 6.55 | 5.74 ; 7.36 | 2,49 | 0,87 | < 0.0001 *** |  |
| Postoperative pain VAS | Total | 25 | 799 | 2.90 | 2.23 ; 3.58 | 1,86 | 0,98 | < 0.0001 *** | 0.8221 |
|  | Open | 8 | 281 | 2.85 | 1.98 ; 3.71 | 1,93 | 0,99 | < 0.0001 *** |  |
|  | Endoscopic | 17 | 518 | 2.94 | 2.17 ; 3.71 | 1,93 | 0,7 | < 0.0001 *** |  |
| Change in pain VAS | Total | 23 | 692 | -3.48 | -4.84 ; -2.12 | 7,36 | 0,99 | < 0.0001 *** | 0.4156 |
|  | Open | 7 | 243 | -2.8 | -4.99 ; -0.61 | 7,51 | 1 | < 0.0001 *** |  |
|  | Endoscopic | 16 | 449 | -3.93 | -5.7 ; -2.15 | 7,51 | 0,53 | 0.0066 ** |  |
