# Supplementary material for: Endoscopic gluteal tendon repair reduces complication rates while achieving outcomes comparable to open repair: A multilevel meta‐analysis
Source: Knee Surg Sports Traumatol Arthrosc. 2026 Jan 31;34(3):1061–80. doi: 10.1002/ksa.70309 (PMC12948349; doi:10.1002/ksa.70309)
Supplement: Supplementary file 37 — Supporting information. [file KSA-34-1061-s014.docx]

**SUPPLEMENTARY TABLES**

Supplementary Table 1: PRISMA checklist

Supplementary Table 2: TITAN checklist

Supplementary Table 3: AMSTAR 2 checklist

Supplementary Table 4: Summary of the postoperative outcome parameters. *MCID: minimal clinically important difference; mHHS: modified Harris Hip Score; iHOT-12: International Hip Outcome Tool-12 Items; HOOS: Hip disability and Osteoarthritis Outcome Score; HOS‐ADL: Hip Outcome Score – Activities of Daily Living; HOS‐SSS: Hip Outcome Score ‐ Sports Subscale; OHS: Oxford Hip Score; VAS; visual analogue score;*

**SUPPLEMENTARY FIGURES**

Supplementary Figure 1: Forest plot of the preoperative functional MCID. *MCID: minimal clinically important difference; SD: standard deviation; CI: confidence interval;*

Supplementary Figure 2: Forest plot of the preoperative pain MCID. *MCID: minimal clinically important difference; SD: standard deviation; CI: confidence interval;*

Supplementary Figure 3: Forest plot of the preoperative HHS. *HHS: Harris Hip Score; SD: standard deviation; CI: confidence interval;*

Supplementary Figure 4: Forest plot of the postoperative mHHS. *mHHS: modified Harris Hip Score; SD: standard deviation; CI: confidence interval;*

Supplementary Figure 5: Forest plot of the preoperative iHOT-12. *iHOT-12: International Hip Outcome Tool – 12 Items; SD: standard deviation; CI: confidence interval;*

Supplementary Figure 6: Forest plot of the preoperative HOS-SSS. *HOS-SSS: Hip Outcome Score – Sports Specific Subscale; SD: standard deviation; CI: confidence interval;*

Supplementary Figure 7: Forest plot of the preoperative HOS-ADL. *HOS-ADL: Hip Outcome Score – Activities of Daily Living; SD: standard deviation; CI: confidence interval;*

Supplementary Figure 8: Forest plot of the preoperative OHS. *OHS: Oxford Hip Score; SD: standard deviation; CI: confidence interval;*

Supplementary Figure 9: Funnel plot of the postoperative functional MCID. *MCID: minimal clinically important difference;*

Supplementary Figure 10: Funnel plot of the change in functional MCID. *MCID: minimal clinically important difference;*

Supplementary Figure 11: Funnel plot of the postoperative pain MCID. *MCID: minimal clinically important difference;*

Supplementary Figure 12: Funnel plot of the change in pain MCID. *MCID: minimal clinically important difference;*

Supplementary Figure 13: Funnel plot of the overall complications.

Supplementary Figure 14: Forest plot of the postoperative iHOT-12. *iHOT-12: International Hip Outcome Tool – 12 Items; SD: standard deviation; CI: confidence interval;*

Supplementary Figure 15: Forest plot of the postoperative HOS-SSS. *HOS-SSS: Hip Outcome Score – Sports Specific Subscale; SD: standard deviation; CI: confidence interval;*

Supplementary Figure 16: Forest plot of the postoperative HOS-ADL. *HOS-ADL: Hip Outcome Score – Activities of Daily Living; SD: standard deviation; CI: confidence interval;*

Supplementary Figure 17: Forest plot of the postoperative HOOS. *HOOS : Hip disability and Osteoarthritis Outcome Score; SD: standard deviation; CI: confidence interval;*

Supplementary Figure 18: Forest plot of the change in mHHS. *mHHS: modified Harris Hip Score; SD: standard deviation; CI: confidence interval;*

Supplementary Figure 19: Forest plot of the change in iHOT-12. *iHOT-12: International Hip Outcome Tool – 12 Items; SD: standard deviation; CI: confidence interval;*

Supplementary Figure 20: Forest plot of the change in HOS-SSS. *HOS-SSS: Hip Outcome Score – Sports Specific Subscale; SD: standard deviation; CI: confidence interval;*

Supplementary Figure 21: Forest plot of the change in HOS-ADL. *HOS-ADL: Hip Outcome Score – Activities of Daily Living; SD: standard deviation; CI: confidence interval;*

Supplementary Figure 22: Forest plot of the change in OHS. *OHS: Oxford Hip Score; SD: standard deviation; CI: confidence interval;*

Supplementary Figure 23: Funnel plot of the postoperative mHHS. *mHHS: modified Harris Hip Score;*

Supplementary Figure 24: Funnel plot of the postoperative iHOT-12. *iHOT-12: International Hip Outcome Tool – 12 Items;*

Supplementary Figure 25: Funnel plot of the postoperative HOS-SSS. *HOS-SSS: Hip Outcome Score – Sports Specific Subscale;*

Supplementary Figure 26: Funnel plot of the postoperative HOS-ADL. *HOS-ADL: Hip Outcome Score – Activities of Daily Living;*

Supplementary Figure 27: Funnel plot of the postoperative HOOS. *HOOS: Hip disability and Osteoarthritis Outcome Score;*

Supplementary Figure 28: Funnel plot of the postoperative OHS. *OHS: Oxford Hip Score;*

Supplementary Figure 29: Funnel plot of the change in mHHS. *mHHS: modified Harris Hip Score;*

Supplementary Figure 30: Funnel plot of the change in HOS-SSS. *HOS-SSS: Hip Outcome Score – Sports Specific Subscale;*

Supplementary Figure 31: Funnel plot of the change in OHS. *OHS: Oxford Hip Score;*

Supplementary Figure 32: Funnel plot of the change in pain VAS. *VAS: Visual Analog Scale;*
